# Efficacy and safety of yaobitong capsules for lumbar disc herniation: a systematic review and meta-analysis

**DOI:** 10.3389/fphar.2026.1815867

**Published:** 2026-06-01

**Authors:** Shi-Ao Wang, Xiong-Jiang Wang, Si-Jin Che, Hong-Liang Tang, Jun Pang

**Affiliations:** 1 FangChengGang Hospital of Guangxi University of Chinese Medicine, Fangchenggang, Guangxi, China; 2 Guangxi University of Chinese Medicine, Nanning, Guangxi, China

**Keywords:** inflammatory cytokines, lumbar disc herniation, meta-analysis, traditional Chinese medicine, visual analogue scale, yaobitong capsules

## Abstract

**Background:**

Lumbar disc herniation (LDH) is a major contributor to the global burden of low back pain. Yaobitong (YBT) capsules are a commercial Chinese polyherbal preparation that has been widely used to treat LDH. However, its efficacy and safety require comprehensive evaluation.

**Methods:**

A comprehensive search was conducted across eight databases, retrieving literature from the inception of each database up to 31 October 2025. Randomized controlled trials (RCTs) evaluating YBT alone or in combination with standard pharmacological therapy were included. Subgroup and sensitivity analyses explored outcome stability. Publication bias was assessed using funnel plots, Egger’s test, and Begg’s rank correlation test. Study quality was evaluated using the Cochrane Risk of Bias (RoB) tool, and evidence certainty was graded using the Grading of Recommendations Assessment, Development and Evaluation (GRADE) approach. Meta-analysis was performed using RevMan 5.3 and R software.

**Results:**

Seventeen RCTs involving 1,752 patients with LDH were included. Compared with standard pharmacological therapy, YBT capsules significantly improved VAS (MD = −1.37, 95% CI: −1.60 to −1.14), ODI (MD = −5.36, 95% CI: −6.56 to −4.16), and JOA scores (MD = 5.21, 95% CI: 3.66–6.75). Additionally, YBT capsules significantly reduced levels of inflammatory cytokines including IL-6, IL-8, and TNF-α, supporting potential anti-inflammatory effects. Adverse events were mild and less frequent in the YBT capsules group (RR = 0.34, 95% CI: 0.18–0.67). Subgroup and sensitivity analyses demonstrated consistent results across varying sample sizes, treatment periods, and regimens, supporting the robustness of the study findings.

**Conclusion:**

YBT capsule-based therapy was associated with reduced pain, improved functional outcomes, and suppressed inflammatory responses in LDH, while exhibiting a favorable short-term safety profile. These findings support YBT capsules as a potentially effective adjunct to conventional therapy. However, most trials were small, single-center studies conducted in China. Large-scale, multicenter RCTs with standardized outcomes are needed to confirm long-term safety and clinical utility.

**Systematic Review Registration:**

https://www.crd.york.ac.uk/PROSPERO/view/CRD420251232067, identifier CRD420251232067.

## Introduction

Lumbar disc herniation (LDH) is one of the most common conditions, characterized by low back pain (LBP) or leg pain, leg weakness, and sensory abnormalities (Lee et al., 2025). In 2020, approximately 619 million people worldwide were affected by LBP, with the Global Burden of Disease Study (GBD) projecting this number to rise to 843 million by 2050 with a growth rate of 36.4% (Ferreira et al., 2023). Despite a slight decline in age-standardized prevalence, the absolute number of affected individuals continues to climb due to population growth and accelerated aging. Incidence increases significantly with age, peaking in the 80–84 age group, with women exhibiting higher prevalence than men across all age brackets (Ferreira et al., 2023). Given the large and aging population affected by LDH, identifying effective and safer treatment options has become an increasingly important clinical priority.

LDH develops from intervertebral disc degeneration. Symptoms arise when the nucleus pulposus ruptures through the annulus fibrosus into the spinal canal or intervertebral foramen (Zhang et al., 2023). Approximately 95% of cases occur at the L4–L5 or L5–S1 segments (Yang et al., 2025). Traditional views emphasized mechanical compression, but modern research has confirmed that the inflammatory response is the core mechanism underlying pain and nerve stimulation. Extruded disc matrix components induce aseptic inflammation, stimulating immune cells to release large amounts of pro-inflammatory factors such as interleukin-1β (IL-1β) and tumor necrosis factor-α (TNF-α) (Yang et al., 2025; Wang et al., 2021; Gong et al., 2025). This leads to nerve edema and pain sensitization, triggering persistent sciatica (Lu et al., 2022; Chen et al., 2024). Furthermore, systemic metabolic inflammation accelerates disc degeneration and may diminish the efficacy of conservative treatments (Duceac Covrig et al., 2025). Therefore, conservative management strategies should integrate anti-inflammatory, analgesic, and microcirculation-enhancing effects rather than relying solely on mechanical decompression.

The standard pharmacological therapy for conservative treatment during the acute phase of LDH primarily employs nonsteroidal anti-inflammatory drugs (NSAIDs) and other medications to alleviate symptoms and reduce inflammatory responses (Yaman et al., 2024). However, safety concerns regarding NSAIDs are particularly prominent. Long-term use or administration to high-risk patients significantly increases the risk of cardiovascular thrombosis, myocardial infarction, stroke, gastrointestinal bleeding, and renal injury (Bindu et al., 2020; Doña et al., 2020). The elderly are the group most profoundly affected by LDH and most prone to severe adverse reactions. This contradiction creates an urgent clinical need for alternative treatments with fewer adverse effects, multiple targets, and suitability for long-term use. At the same time, traditional Chinese medicine (TCM) has garnered attention due to its safety profile and multi-mechanism advantages. The 2024 Evidence-Based Guidelines for Traditional Chinese Medicine Treatment of Lumbar Disc Herniation recommend interventions including acupuncture, massage therapy, and proprietary Chinese medicines such as Yaobitong (YBT) (Qin et al., 2024). YBT’s inclusion in authoritative guidelines signifies its transition from empirical application to evidence-based medicine. Consequently, a systematic and standardized meta-analysis of its efficacy and safety is warranted.

In summary, we conducted a systematic review and meta-analysis incorporating randomized controlled trials to evaluate the efficacy and safety of YBT capsules in treating LDH. This study aims to provide higher quality evidence to inform evidence-based guidelines and clinical practice, while promoting the standardized and evidence-based application of TCM in the management of modern lumbar disc herniation.

## Materials and methods

This systematic review and meta-analysis was conducted according to the Cochrane Handbook for Systematic Reviews of Interventions and the Preferred Reporting Items for Systematic Reviews and Meta-Analyses (PRISMA) Statement 2020 (Page et al., 2021). The complete PRISMA checklist is shown in Supplementary Table S1. The study protocol has been registered in the International Prospective Register of Systematic Reviews (PROSPERO) (registration number CRD420251232067).

### Databases and search strategies

A systematic literature search was conducted in English and Chinese databases, including PubMed, Embase, Web of Science, Cochrane Library, China National Knowledge Infrastructure (CNKI), Wanfang, China Biology Medicine disc (CBM) and VIP. The search scope covered all databases from their inception to 31 October 2025. The search strategy combined subject headings with free-text terms, including keywords such as “Yaobitong,” “lumbar disc herniation,” “intervertebral disc,” “low back pain,” and “randomized controlled trial.” AND/OR logical operators were used to enhance search sensitivity and specificity. Additionally, manual hand-searching of reference lists from relevant publications was conducted. No language restrictions were applied during the initial screening phase. The complete search terms for each database are provided in the Supplementary Table S2.

### Description of yaobitong capsules

YBT capsules are a commercial proprietary Chinese medicinal product used for the conservative treatment of LDH. YBT capsules contain Notoginseng Radix et Rhizoma (San Qi), derived from *Panax notoginseng* (Burkill) F.H.Chen [Araliaceae]; Chuanxiong Rhizoma (Chuan Xiong), derived from *Ligusticum chuanxiong* Hort [Apiaceae]; Corydalis Rhizoma (Yan Hu Suo), derived from *Corydalis yanhusuo* W.T.Wang ex Z.Y.Su and C.Y.Wu [Papaveraceae]; Paeoniae Radix Alba (Bai Shao), derived from *Paeonia lactiflora* Pall [Paeoniaceae]; Achyranthis Bidentatae Radix (Niu Xi), derived from *Achyranthes bidentata* Blume [Amaranthaceae]; Cibotii Rhizoma (Gou Ji), derived from *Cibotium barometz* (L.) J. Sm [Cyatheaceae]; Rhei Radix et Rhizoma (Shu Da Huang), derived from *Rheum palmatum* L., *Rheum tanguticum* (Maxim. ex Regel) Balf., or *Rheum officinale* Baill [Polygonaceae]; and Angelicae Pubescentis Radix (Du Huo), derived from *Angelica biserrata* (R.H.Shan and C.Q.Yuan) C.Q.Yuan and R.H.Shan [Apiaceae]. YBT capsules are manufactured by Jiangsu Kangyuan Pharmaceutical Co., Ltd., with a reported specification of 0.42 g per capsule and Chinese drug approval number Z20010,045. The recommended dosage for YBT capsules is three times daily, 3 capsules each time, taken orally after meals. Adverse reactions reported by the manufacturer include gastrointestinal symptoms such as nausea, vomiting, abdominal pain, diarrhea, and stomach discomfort. Other reported adverse reactions include headache, dizziness, itching, and rash. This medication is contraindicated in pregnant women and patients with hypersensitivity to this product or any of its ingredients. The manufacturer’s instructions also state that YBT should not be used in combination with Veratri Rhizoma et Radix (Li Lu). In the included randomized controlled trials (RCTs), YBT capsules were used either alone or as an adjunctive intervention for LDH. However, because this meta-analysis synthesizes published clinical reports, and the original trials did not consistently report detailed information regarding inter-batch quality attributes, analytical certificates, excipients, processing methods, extraction procedures, and product-specific phytochemical fingerprints, a comprehensive evaluation could not be performed. A summary of the reported information on the YBT product from each included study is presented in Supplementary Table S3.

### Inclusion criteria

Inclusion criteria are as follows:

1. Study type: RCTs;
2. Study subjects: Patients diagnosed with symptomatic LDH based on imaging studies combined with corresponding clinical manifestations or established diagnostic criteria, regardless of age or gender;
3. Intervention: YBT capsules alone or in combination with standard pharmacological therapy or physical therapy;
4. Control measures: Placebo, no treatment, or standard pharmacological therapy/physical therapy;
5. Outcome measures: Report at least one clinically relevant outcome, including the primary outcome visual analogue scale (VAS), secondary outcomes Oswestry Disability Index (ODI), Japanese Orthopaedic Association Score (JOA), interleukin-6 (IL-6), interleukin-8 (IL-8), tumor necrosis factor-α (TNF-α), or adverse events;
6. Studies providing sufficient data for effect size calculation.

### Exclusion criteria

Exclusion criteria are as follows:

1. Observational studies, case reports, case series, reviews or conference abstracts, animal studies, *in vitro* research, or other non-clinical studies.
2. Studies in which additional Chinese herbal formulations or non-equivalent co-interventions were added only to the experimental arm, while the control group did not receive comparable treatment, precluding an independent assessment of the effect of YBT capsules.
3. Duplicate publications or studies with redundant data, only the most comprehensive or latest version will be included.

### Data extraction and quality assessment

Two researchers independently performed literature screening, data extraction, and methodological quality assessment using the Cochrane Risk of Bias (RoB) Tool. Extracted data included study basic information (first author, publication year, sample size), subject characteristics (age, gender ratio), treatment interventions, treatment duration, disease duration, VAS scale format, and funding or manufacturer involvement where reported. The duration of the disease is defined as the time elapsed since the radiological diagnosis of LDH. Symptom duration from onset was not reported in the included studies. Clinical outcome measures collected included VAS, ODI, JOA, IL-6, IL-8, TNF-α, and adverse events. If the study information was incomplete, attempts were made to contact the authors for further details. Disagreements during screening or data extraction were resolved through discussion or consultation with a third researcher.

### Certainty assessment of evidence

This study assessed the quality of evidence for primary and secondary outcomes based on the Grading of Recommendations Assessment, Development and Evaluation (GRADE) methodological framework. Two investigators independently rated the evidence in terms of five dimensions: risk of bias, inconsistency, indirectness, imprecision, and publication bias, and categorized the evidence as high, moderate, low, and very low. All included studies were RCTs and therefore initially rated as high, but were downgraded if there were deficiencies in study quality or statistical results. Disagreements during the rating process, if any, were adjudicated through discussion or by inviting a third investigator. A quality of evidence summary table was generated using GRADEpro GDT software.

### Statistical analysis

Binary outcomes were pooled using risk ratios (RR) with 95% confidence intervals (95% CIs). Continuous outcomes were analyzed using mean differences (MD) or standardized mean differences (SMD) based on scale characteristics. Due to inconsistent measurement units for inflammatory markers (e.g., IL-6, IL-8, TNF-α) across studies, SMDs were uniformly applied for meta-analysis to ensure comparability of effect sizes. When multiple time points were reported, we extracted the measurement at the end of the planned treatment period as the primary endpoint for analysis. Heterogeneity was assessed using the *I* ^*2*^ statistic and by considering clinical and methodological differences across studies. Given the expected between-study variability in treatment regimens, co-interventions, treatment duration, and outcome assessment, a random-effects model was applied when heterogeneity was substantial, whereas a fixed-effect model was used when heterogeneity was low and the studies were considered sufficiently comparable. For the primary endpoint VAS, we conducted subgroup analyses based on treatment duration, sample size, treatment regimen, disease duration, and mean age to explore potential sources of heterogeneity and assess consistency of treatment effects across clinically relevant stratifications. Sensitivity analysis was conducted by leave-one-out method to assess the robustness of pooled results. When ≥10 studies reported a specific outcome, funnel plots, Egger’s regression test, and Begg’s rank correlation test were used to detect publication bias. All statistical analyses were performed using RevMan 5.3 and R software (version 4.3.2, “meta” package).

## Results

### Study selection

**FIGURE 1:**
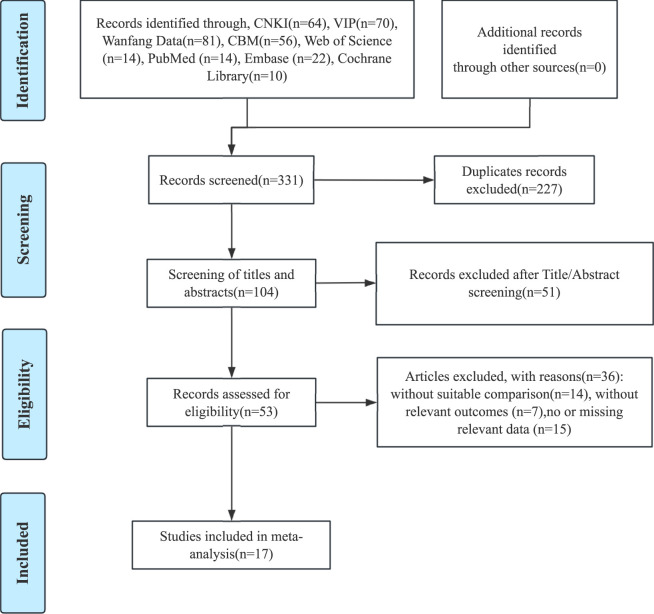
PRISMA flow diagram of study selection. Flow diagram showing the identification, screening, eligibility assessment, and final inclusion of studies in the meta-analysis. PRISMA, Preferred Reporting Items for Systematic Reviews and Meta-Analyses.

A total of 331 studies were retrieved from sources including CNKI, VIP, Wanfang, CBM, Web of Science, PubMed, Embase, and the Cochrane Library. After removing 227 duplicate studies, 104 studies underwent title and abstract screening. Among these, 51 were excluded due to irrelevance to the research topic or being non-clinical studies. A total of 53 full-text articles were evaluated. Thirty-six were excluded due to lack of an appropriate control group (n = 14), failure to report relevant outcomes (n = 7), or missing relevant data (n = 15). Ultimately, 17 RCTs were included in the meta-analysis. The study selection process is illustrated in Figure 1.

### Study characteristics

This study included 17 RCTs (Wang and Ao, 2025; Li and Wang, 2025; Yan, 2022; Xin et al., 2021; Zhong and Luo, 2019; Wang et al., 2018; Su et al., 2018; Ma, 2018; Wu, 2017; Wang et al., 2017; Dong et al., 2017; Wu et al., 2015; Sheng, 2015; Shao, 2015; Gao, 2015; Hu et al., 2014; Zhou and Meng, 2012) involving a total of 1,752 patients with LDH (Table 1). The sample sizes of the included studies ranged from 36 to 152 participants. The mean age of participants was predominantly between 32 and 60 years, with age and gender distributions generally consistent across trial groups. Treatment duration varied from 2 weeks to 1 month. For the VAS outcome, the extracted scores across trials were consistent with a 0–10 point scale, as documented in Table 1. All studies employed YBT capsules, typically administered at a dose of 3 capsules three times daily. Most trials employed YBT capsules as monotherapy. A minority of trials combined YBT with standard pharmacological therapy such as NSAIDs (celecoxib, loxoprofen, ibuprofen, diclofenac, naproxen, or meloxicam) and/or physical treatments including lumbar traction, massage, acupuncture, and TDP therapy. The control group received the corresponding standard treatment without YBT capsules. In all included RCTs, YBT capsules were supplied as the same proprietary product with a uniform specification. The dosage form and strength of the capsules were consistent across studies, while only the total daily dose and treatment duration varied between trials, as detailed in Table 1. This reduces variability related to formulation differences and allows a more direct comparison of clinical effects across studies. Overall, the comparison was between YBT-based therapy plus standard treatment versus standard treatment alone, with well-balanced baseline characteristics.

**TABLE 1:** Characteristics of included randomized controlled trials.

Study ID	Sample size		Age (Year)		Male/Female		Disease duration	Duration of treatment	VAS scale	Funding/Manufacturer involvement	Intervention		Outcomes
	T	C	T	C	T	C					Treatment	Control	
Wang and Ao (2025)	50	50	60.94 ± 5.22	61.16 ± 4.90	31/19	29/21	T: 2–5 years, 3.12 ± 0.69; C: 2–5 years, 3.28 ± 0.31	1 month	0–10 points	NR	(1) YBT capsule, 3 capsules three times daily (2) Celecoxib 200 mg once daily	Celecoxib 200 mg once daily	①②④⑥⑦
Li and Wang (2025)	40	40	54.71 ± 3.85	53.26 ± 4.12	22/18	21/19	T: 6–15 months, 11.03 ± 2.15; C: 6–15 months, 10.24 ± 2.03	2 weeks	0–10 points	NR	(1) YBT capsule, 3 capsules three times daily (2) celecoxib 200 mg once daily (3) intravenous infusion of ketorolac tromethamine 60 mg per dose, once daily	(1) celecoxib 200 mg once daily (2) intravenous infusion of ketorolac tromethamine 60 mg per dose, once daily	①②③④⑥⑦
Yan (2022)	60	60	42.02 ± 6.14	40.62 ± 4.34	38/22	40/20	T: 1–8 weeks, 6.38 ± 1.75; C: 1–8 weeks, 6.43 ± 1.84	2 weeks	0–10 points	NR	(1) YBT capsule, 3 capsules three times daily (2) Lumbar traction and manual therapy	(1) Loxoprofen sodium capsules, 60 mg per capsule, three times daily (2) Lumbar traction and manual therapy	①④⑤⑦
Xin et al. (2021)	48	48	45.82 ± 2.01	45.77 ± 2.06	21/27	24/24	T: 6 months–3 years, 1.48 ± 0.72 years; C: 6 months–3 years, 1.52 ± 0.69 years	1 month	0–10 points	Shandong traditional Chinese medicine science and technology development plan, no. 2019–0,111; manufacturer involvement: NR	(1) YBT capsule, 3 capsules three times daily (2) celecoxib, 200 mg once daily	Celecoxib 200 mg once daily	①②④
Zhong and Luo (2019)	48	48	58.14 ± 6.43	58.36 ± 6.29	29/19	23/19	NR	1 month	0–10 points	NR	(1) YBT capsule, 3 capsules three times daily (2) mannitol 250 mL mixed with dexamethasone 15 mg, intravenous drip once daily	Mannitol 250 mL mixed with dexamethasone 15 mg, intravenous drip once daily	①②
Wang et al. (2018)	66	66	22–73	23–77	34/32	40/26	NR	1 month	NR	NR	(1) YBT capsule, 3 capsules three times daily	Naproxen sustained-release capsules, 0.5 g per dose, once daily	①
Su et al. (2018)	42	42	26–57	25–54	29/13	26/16	T: 1–42 months, 10.81 ± 4.32; C: 1.5–39 months, 11.71 ± 4.65	4 weeks	NR	NR	(1) YBT capsule, 3 capsules three times daily (2) Lumbar traction and standardized manual therapy	(1) Loxoprofen sodium tablets, 60 mg per dose, three times daily (2) Lumbar traction and standardized manual therapy	①④
Ma (2018)	72	72	54.8 ± 6.4	55.7 ± 6.3	33/39	32/40	T: 11–58 days, 32.1 ± 4.7; C: 10–60 days, 31.2 ± 4.6	2 weeks	0–10 points	NR	(1) YBT capsule, 3 capsules three times daily (2) Lumbar traction and manual therapy	(1) Loxoprofen sodium capsules, 1 capsule per dose, three times daily (2) Lumbar traction and manual therapy	①④⑤⑦
Wu (2017)	40	40	50.2 ± 5.3	49.3 ± 4.4	23/17	22/18	T: 3–13 months, 7.3 ± 0.8; C: 1–11 months, 5.6 ± 0.2	4 weeks	Not applicable	NR	(1) YBT capsule, 3 capsules three times daily (2) Routine therapies including traction, massage, and acupuncture	(1) Ibuprofen sustained-release capsules, 1 capsule per dose, twice daily (2) Routine therapies including traction, massage, and acupuncture	⑥
Wang (2017)	76	76	47.01 ± 2.89	45.23 ± 3.37	30/46	36/40	T: 10–20 years, 16.66 ± 3.35; C: 10–20 years, 15.28 ± 2.55	4 weeks	NR	NR	(1) YBT capsule, 3 capsules three times daily (2) conventional physiotherapy including TDP irradiation, acupuncture, massage, and lumbar traction	(1) meloxicam capsules, 1 capsule per dose, twice daily (2) conventional physiotherapy including TDP irradiation, acupuncture, massage, and lumbar traction	①③⑦
Dong et al. (2017)	47	47	52.4 ± 4.5	53.2 ± 4.6	28/19	29/18	T: 1–16 months, 7.0 ± 1.4; C: 1–18 months, 6.9 ± 1.4	4 weeks	0–10 points	NR	(1) YBT capsule, 3 capsules three times daily (2) diclofenac sodium enteric-coated tablets, 50 mg per dose, three times daily (3) mannitol 250 mL with 20% concentration, once daily	(1) diclofenac sodium enteric-coated tablets, 50 mg per dose, three times daily (2) mannitol 250 mL with 20% concentration, once daily	①③④⑥⑦
Wu et al. (2015)	60	60	56.23 ± 16.23	57.14 ± 17.45	24/36	25/30	NR	2 weeks	0–10 points	NR	(1) YBT capsule, 3 capsules three times daily (2) Lumbar traction and massage therapy	(1) Loxoprofen sodium capsules, 1 capsule per dose, three times daily (2) Lumbar traction and massage therapy	①④⑤⑦
Sheng (2015)	18	18	38.91 ± 5.47	38.55 ± 5.17	10/8	11/7	T: 1–8 years, 2.14 ± 0.55; C: 1–8.3 years, 2.52 ± 0.57	1 month	0–10 points	NR	YBT capsule, 3 capsules three times daily	Ibuprofen sustained-release capsules, 1 capsule per dose, twice daily	①
Shao (2015)	43	43	40.3 ± 10.8	39.8 ± 11.3	27/16	25/18	T: 3.2 ± 1.3 years; C: 3.8 ± 1.9 years	4 weeks	0–10 points	NR	(1) YBT capsule, 3 capsules three times daily (2) Lumbar traction, massage, and acupuncture	(1) Ibuprofen sustained-release capsules, 300 mg per capsule, twice daily (2) Lumbar traction, massage, and acupuncture	①
Gao (2015)	61	61	32 ± 8	32 ± 8	32/29	30/31	T: 1–9 years, 6.0 ± 1.6; C: 1–10 years, 5.0 ± 1.3	1 month	0–10 points	NR	YBT capsule, 3 capsules twice daily	Naproxen sustained-release capsules 0.5 g once daily	①
Hu et al. (2014)	48	48	40–60	40–60	—	—	T: 10–65 days, 30.0 ± 18.5 days; C: 10–65 days, 31.6 ± 20.0 days	2 weeks	0–10 points	NR	(1) YBT capsule, 3 capsules per dose, three times daily (2) Lumbar traction	(1) Eperisone hydrochloride tablets, 1 tablet per dose, three times daily (2) Loxoprofen sodium capsules, 1 capsule per dose, three times daily (3) Lumbar traction	①
Zhou and Meng (2012)	57	57	32.1 ± 8.35	33.2 ± 8.41	36/21	34/23	T: 1–10 years, 2.3 ± 2.51; C: 1–10 years, 2.2 ± 2.39	4 weeks	NR	NR	(1) YBT capsule, 3 capsules per dose, three times daily (2) Lumbar traction and manual therapy	(1) naproxen sustained-release capsules, 0.5 g per dose, once daily (2) Lumbar traction and manual therapy	①⑥

C, control group; T, intervention group; YBT, yaobitong; NR, not reported. ①, Visual analogue scale; ②, Oswestry Disability Index; ③, Japanese Orthopaedic Association Score; ④, Interleukin-6; ⑤, Interleukin-8; ⑥, Tumor necrosis factor-α; ⑦, Adverse events. Disease duration is presented as range and/or mean ± standard deviation for the treatment and control groups, according to the original studies.

### Quality evaluation

**FIGURE 2:**
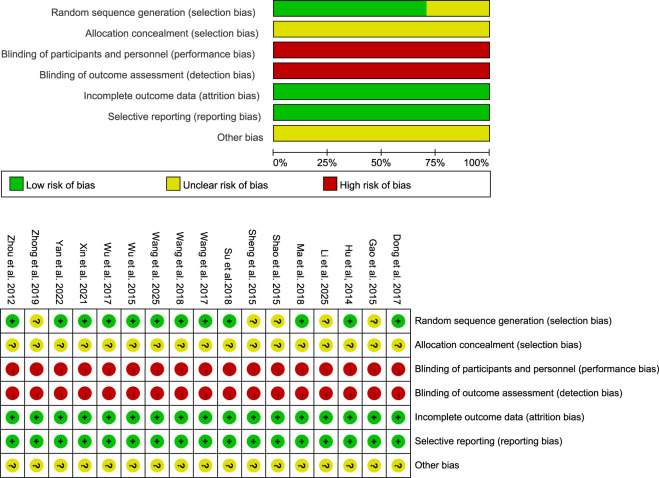
Risk of bias assessment for included randomized controlled trials. Overall and study-level risk of bias assessed using the original Cochrane Risk of Bias tool. Green indicates low risk of bias, yellow indicates unclear risk of bias, and red indicates high risk of bias. Domains assessed included random sequence generation, allocation concealment, blinding, incomplete outcome data, selective reporting, and other bias.

The 17 RCTs included in this study were evaluated according to the Cochrane RoB tool (Figure 2). Twelve of the studies clearly stated the method of generating the randomized sequence (random number table, computerized random numbers, or lottery method) and were rated as low risk. Another five studies mentioned only “randomized groups” but did not specify the method and were rated as unclear risk. Due to insufficient details regarding allocation concealment procedures, the risk of bias for this domain was assessed as unclear across all studies. Regarding the implementation of blinding, none of the included studies explicitly reported effective blinding procedures. Given the significant differences between YBT-based treatment and control treatments in terms of administration frequency, route of administration, and treatment protocols, implementing blinding was considered difficult or impractical in many trials. Therefore, all included studies were rated as high risk for the blinding-related domains. Seventeen studies reported the prespecified outcome indicators, and no clear selective reporting or obvious baseline imbalances were identified. However, detailed information on withdrawals and handling of missing data was rarely provided, so attrition bias cannot be confidently excluded. Although no obvious baseline imbalance was identified, information on funding and manufacturer involvement was generally insufficiently reported. Consequently, the “other bias” domain was judged as unclear risk for all included studies. Overall, the main risk of bias coming from allocation concealment and inadequate blinding.

### VAS

**FIGURE 3:**
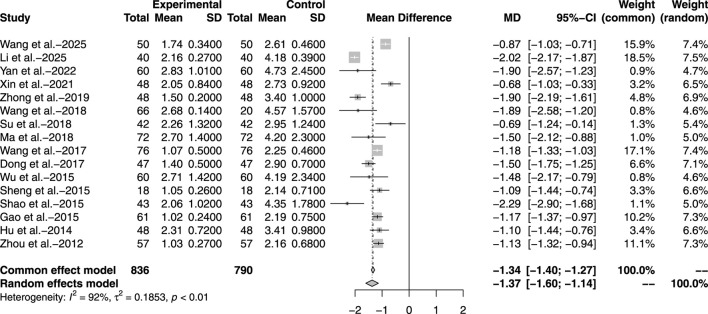
Forest plot of VAS in patients with lumbar disc herniation. Forest plot comparing YBT capsule-based therapy with the corresponding conventional non-YBT treatment in patients with lumbar disc herniation. Effect size is presented as MD with 95% CI using a random-effects model. Negative values favor YBT. VAS, visual analogue scale; YBT, Yaobitong; MD, mean difference; CI, confidence interval.

A total of 16 RCTs reported VAS involving 1,626 patients with LDH (Figure 3). High heterogeneity was observed in the results (*I* ^*2*^ = 92%, *p* < 0.01), thus a random-effects model was applied. Meta-analysis results indicated that compared with the control group, interventions based on YBT capsules significantly reduced VAS scores, with a statistically significant difference (MD = −1.37, 95% CI: −1.60 to −1.14). Sensitivity analyses confirmed the robustness and reliability of these findings (Supplementary Figure S1).

### ODI

**FIGURE 4:**
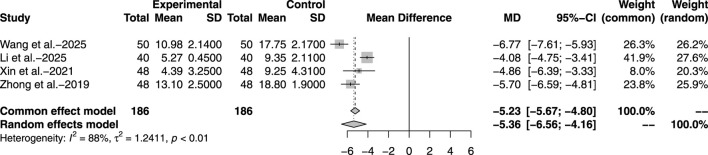
Forest plot of ODI in patients with lumbar disc herniation. Forest plot comparing YBT capsule-based therapy with the corresponding conventional non-YBT treatment in patients with lumbar disc herniation. Effect size is presented as MD with 95% CI using a random-effects model. Negative values favor YBT. ODI, Oswestry Disability Index; YBT, Yaobitong; MD, mean difference; CI, confidence interval.

Four RCTs reported ODI involving 372 patients with LDH (Figure 4). Due to high heterogeneity (*I* ^*2*^ = 88%, *p* < 0.01), analysis was conducted using a random-effects model. The meta-analysis revealed that YBT capsule based interventions significantly reduced ODI scores compared to the control group (MD = −5.36, 95% CI: −6.56 to −4.16). Sensitivity analysis indicates that effect sizes remain stable after excluding individual studies, suggesting robust results (Supplementary Figure S2).

### JOA

**FIGURE 5:**
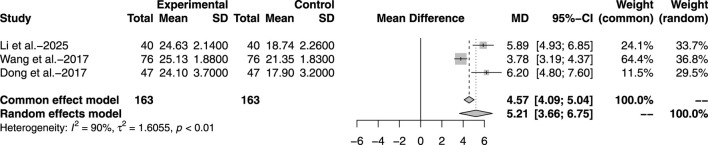
Forest plot of JOA score in patients with lumbar disc herniation. Forest plot comparing YBT capsule-based therapy with the corresponding conventional non-YBT treatment in patients with lumbar disc herniation. Effect size is presented as MD with 95% CI using a random-effects model. Positive values favor YBT. JOA, Japanese Orthopaedic Association; YBT, Yaobitong; MD, mean difference; CI, confidence interval.

Three studies reported the JOA scores for YBT capsules in treating LDH (Figure 5). Due to significant heterogeneity (*I* ^*2*^ = 90%, *p* < 0.01), a random-effects model was selected for statistical analysis. Results indicated that compared with conventional drug therapy, YBT capsules effectively improved JOA scores in patients with LDH, with statistically significant differences (MD = 5.21, 95% CI: 3.66–6.75). Sensitivity analysis revealed no significant changes in the pooled effect size after excluding any individual study, indicating robust results (Supplementary Figure S3).

### IL-6

**FIGURE 6:**
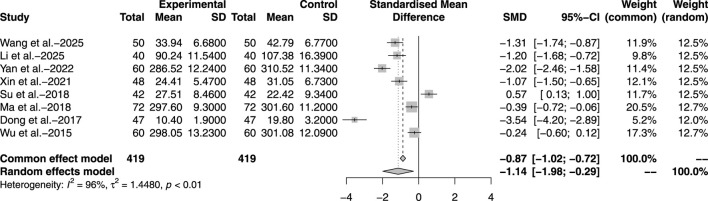
Forest plot of IL-6 in patients with lumbar disc herniation. Forest plot comparing YBT capsule-based therapy with the corresponding conventional non-YBT treatment in patients with lumbar disc herniation. Effect size is presented as SMD with 95% CI using a random-effects model. Negative values favor YBT. IL-6, interleukin-6; YBT, Yaobitong; SMD, standardized mean difference; CI, confidence interval.

A total of eight studies reported the IL-6 level of YBT capsules in treating LDH (Figure 6). Based on the heterogeneity test (*I* ^*2*^ = 96%, *p* < 0.01), a random-effects model was selected for statistical analysis. The results showed that compared with standard pharmacological therapy, YBT capsules effectively reduced IL-6 levels in patients with LDH, with a statistically significant difference (SMD = −1.14, 95% CI: −1.98 to −0.29). Sensitivity analysis demonstrates stable results (Supplementary Figure S4).

### IL-8

**FIGURE 7:**
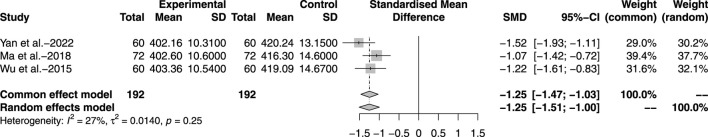
Forest plot of IL-8 in patients with lumbar disc herniation. Forest plot comparing YBT capsule-based therapy with the corresponding conventional non-YBT treatment in patients with lumbar disc herniation. Effect size is presented as SMD with 95% CI using a fixed-effect model. Negative values favor YBT. IL-8, interleukin-8; YBT, Yaobitong; SMD, standardized mean difference; CI, confidence interval.

For the IL-8 outcome, a total of 3 RCTs were included (Figure 7). Due to low heterogeneity (*I* ^*2*^ = 27%, *p =* 0.25), a fixed-effect model was used for the pooled analysis. Results showed that compared with standard pharmacological therapy, YBT capsules significantly reduced IL-8 levels (SMD = −1.25, 95% CI: −1.47 to −1.03). Sensitivity analysis indicates that effect sizes remain stable after excluding individual studies, suggesting robust results (Supplementary Figure S5).

### TNF-α

**FIGURE 8:**
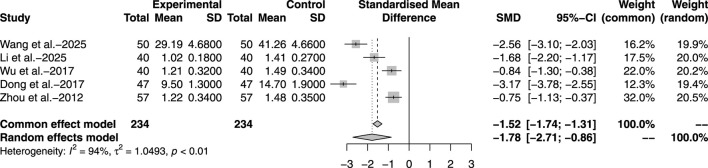
Forest plot of TNF-α in patients with lumbar disc herniation. Forest plot comparing YBT capsule-based therapy with the corresponding conventional non-YBT treatment in patients with lumbar disc herniation. Effect size is presented as SMD with 95% CI using a random-effects model. Negative values favor YBT. TNF-α, tumor necrosis factor-α; YBT, Yaobitong; SMD, standardized mean difference; CI, confidence interval.

A total of five studies reporting TNF-α outcomes were included (Figure 8). Due to significant heterogeneity (*I* ^*2*^ = 94%, *p* < 0.01), a random-effects model was used for meta-analysis. Results showed that compared with standard pharmacological therapy, YBT capsules significantly reduced TNF-α levels (SMD = −1.78, 95% CI: −2.71 to −0.86). Sensitivity analyses confirmed the robustness of these findings (Supplementary Figure S6).

### Adverse events

**FIGURE 9:**
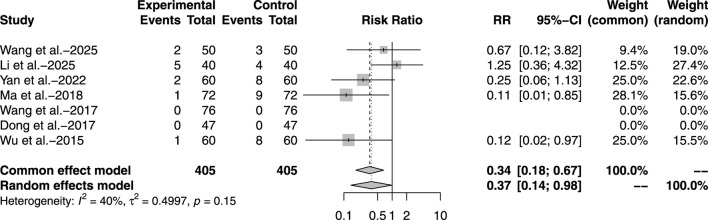
Forest plot of adverse events in patients with lumbar disc herniation. Forest plot comparing YBT capsule-based therapy with the corresponding conventional non-YBT treatment in patients with lumbar disc herniation. Effect size is presented as RR with 95% CI using a fixed-effect model. Values below 1 favor YBT, indicating fewer adverse events in the YBT group than in the corresponding conventional treatment group. YBT, Yaobitong; RR, risk ratio; CI, confidence interval.

A total of seven studies reported adverse events (Figure 9). Due to low heterogeneity (*I* ^*2*^ = 40%, *p* = 0.15), a fixed-effect model was used for analysis. Meta-analysis results indicated that the incidence of adverse reactions in the YBT capsule group was lower than that in the control group (RR = 0.34, 95% CI: 0.18–0.67). Adverse events reported included mild gastrointestinal discomfort, dizziness, and mild rash. No serious drug-related adverse events were observed. Sensitivity analyses confirmed the stability and reliability of the results (Supplementary Figure S7).

### Subgroup analysis

To validate the robustness of the primary analysis results, subgroup analyses were conducted based on treatment duration, sample size, treatment regimen, disease duration, and mean age. After grouping by treatment course, the 2 weeks (MD = −1.61, 95% CI: −2.00 to −1.22), 4 weeks (MD = −1.33, 95% CI: −1.77 to −0.89), and 1 month (MD = −1.23, 95% CI: −1.63 to −0.84) subgroups all demonstrated that YBT capsules significantly outperformed the control in improving VAS scores, suggesting that efficacy is not limited by treatment duration (Supplementary Figure S8).

After grouping by sample size, YBT capsules significantly improved VAS scores compared to the control group in the small (n ≤ 80, MD = −1.69, 95% CI: −2.25 to −1.12), medium (81 ≤ n ≤ 120, MD = −1.28, 95% CI: −1.56 to −1.00), and large sample groups (n > 120, MD = −1.20, 95% CI: −1.39 to −1.01), indicating that study scale had limited impact on the results (Supplementary Figure S9).

After grouping by treatment regimen, both YBT capsules alone (MD = −1.32, 95% CI: −1.55 to −1.09) and YBT capsules combined with NSAIDs (MD = −1.28, 95% CI: −1.88 to −0.68) demonstrated significantly greater efficacy in improving VAS outcomes compared to the NSAID group (Supplementary Figure S10).

After grouping by disease duration, YBT capsules significantly improved VAS scores compared to the control group in both the >12 months subgroup (MD = −1.16, 95% CI: −1.53 to −0.80) and the ≤12 months subgroup (MD = −1.41, 95% CI: −1.75 to −1.06), suggesting that efficacy was not substantially influenced by disease duration (Supplementary Figure S11). However, the between-subgroup difference was not statistically significant under the random-effects model.

After grouping by mean age, YBT capsules significantly improved VAS scores compared to the control group in both the ≥55 years subgroup (MD = −1.42, 95% CI: −1.91 to −0.92) and the <55 years subgroup (MD = −1.41, 95% CI: −1.73 to −1.08), suggesting that the observed benefit was generally consistent across studies with relatively older or younger participant profiles (Supplementary Figure S12). Similarly, for adverse events, the overall direction also favored YBT in both subgroups, and a more pronounced reduction in reported adverse events was observed in the ≥55 years subgroup (RR = 0.23, 95% CI: 0.07–0.79) (Supplementary Figure S13). However, the between-subgroup difference was not statistically significant.

Overall, subgroup analyses demonstrated consistent efficacy across varying treatment durations, sample sizes, treatment regimens, disease duration, and mean age, with no clear evidence that these factors materially modified the treatment effect, supporting the robustness of the pooled results.

### Sensitivity analysis

To further assess the robustness of the primary outcome measure, we conducted a sensitivity analysis excluding studies with unclear randomization methods (Supplementary Figure S14). After exclusion, the pooled effect remained statistically significant under the random-effects model (MD = −1.20, 95% CI: −1.42 to −0.98, *p* < 0.0001). Although heterogeneity was reduced compared to the original analysis, it remained quite substantial (*I* ^*2*^ = 75.1%), suggesting that unclear reporting of randomization may be a partial, but not the sole, contributor to the observed heterogeneity. Importantly, the direction and magnitude of the pooled effects were generally consistent with the primary analysis results, indicating that the primary outcomes are robust and not driven by studies with unclear reporting of randomization methods.

### Publication bias assessment

**FIGURE 10:**
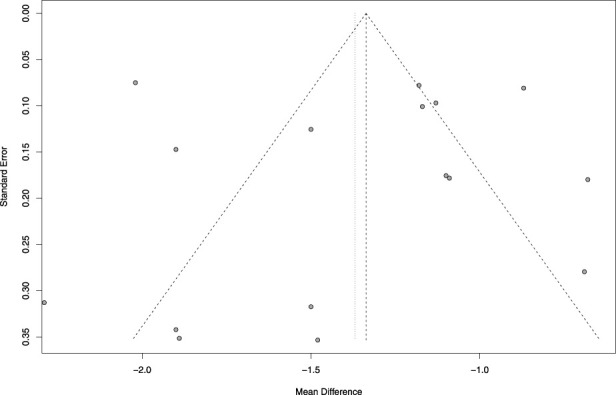
Funnel plot for publication bias assessment of the VAS outcome. Funnel plot of studies included in the VAS meta-analysis comparing YBT capsule-based therapy with the corresponding conventional non-YBT treatment, used to assess potential publication bias or small-study effects. A more symmetrical distribution suggests less evidence of publication bias, whereas marked asymmetry may indicate possible small-study effects or reporting bias. VAS, visual analogue scale; YBT, Yaobitong.

For outcomes with ≥10 included studies, publication bias was assessed using funnel plots, Egger’s test, and Begg’s test for rank correlation. For the VAS outcome, funnel plots exhibited overall symmetrical distribution with no apparent small-sample effect (Figure 10). Egger’s regression test showed no statistical evidence of funnel plot asymmetry (t = −0.17, df = 14, *p* = 0.87), and Begg’s rank correlation test also yielded non-significant results (z = −0.81, *p* = 0.42). These findings suggest no clear evidence of small-study or publication bias for the VAS outcome. However, given the limited number and single-country origin of the trials, the presence of some small-study effects cannot be completely excluded.

### Certainty assessment

According to the GRADE assessment, the quality of evidence ranged from moderate to very low (Supplementary Table S4). Downgrading was mainly related to insufficient reporting of allocation concealment, lack of effective blinding, limited information on attrition or missing data, and substantial heterogeneity in several pooled outcomes. These limitations underscore the need for standardized approaches in large-scale, rigorously designed randomized controlled trials to confirm the efficacy and safety of YBT capsules for treating LDH.

## Discussion

This study included 17 RCTs involving 1,752 patients. Results showed that YBT capsules, whether used alone or in combination with standard pharmacological therapy, demonstrated significantly greater improvements in VAS, ODI, and JOA scores compared to the control group. These findings suggest that YBT capsules may help relieve pain and improve functional impairment in patients with LDH. YBT capsules also reduced inflammatory mediators such as IL-6, IL-8, and TNF-α, supporting potential anti-inflammatory effects. Although heterogeneity was substantial for some outcomes, subgroup and sensitivity analyses supported the overall stability of the main findings. Regarding safety, the available evidence suggested favorable short-term tolerability, with reported adverse events being mostly mild and reversible, and no serious drug-related adverse events described in the included studies. Overall, YBT capsules may represent a potentially useful conservative option for pain relief, functional improvement, and inflammation control in LDH. However, given the methodological limitations and high heterogeneity of these studies, their findings should be regarded as preliminary conclusions rather than definitive ones.

YBT capsules are a commercial Chinese polyherbal preparation widely used for the conservative treatment of LDH in China (Qin et al., 2024). Its therapeutic effect on LDH is believed to stem from multi-targeted pharmacological actions involving anti-inflammatory, analgesic, antioxidant, and neuroprotective mechanisms. The pathogenesis of LDH is closely associated with the release of inflammatory mediators, neural sensitization, and microcirculatory dysfunction (Wang et al., 2021). IL-6, IL-8, and TNF-α are key cytokines that amplify local inflammatory cascades, activate pain signaling pathways, promote proteoglycan degradation, and accelerate disc degeneration (Zhou et al., 2016). Our meta-analysis demonstrated that YBT significantly reduced serum levels of these cytokines, indicating that the capsule alleviates symptoms such as pain and improves functional impairment by suppressing the inflammatory response. These findings are consistent with experimental and omics research results. *In vitro* and *in vivo* studies indicate that YBT downregulates the expression of IL-1β, IL-6, TNF-α, and IL-8 while inhibiting the activation of NF-κB and COX-2 signaling pathways, thereby blocking the initiation and amplification of inflammatory responses (Zhao et al., 2024; Shi et al., 2022). Furthermore, animal studies reveal that YBT capsules ameliorate symptoms and nerve root lesions in rat autologous nucleus pulposus transplantation models by inhibiting p38 MAPK phosphorylation, suggesting its potential to alleviate nerve root inflammation and hyperalgesia (Xin et al., 2015). Pharmacological network analysis has identified 56 bioactive constituents in YBT capsules, such as ginsenoside Rg1, ginsenoside Rb1, senkyunolide H, and tetrahydropalmatine. These active ingredients act on 87 target genes, regulating 29 signaling pathways including MAPK, Ras, PI3K/Akt, and NF-κB. By suppressing excessive inflammatory responses, they help reduce neural hypersensitivity and pain, thereby alleviating LDH-related symptoms (Deng et al., 2020). Furthermore, recent metabolomics studies suggest that YBT may exert broader immunometabolic regulatory effects. By modulating phosphatidylcholine, bile acids, and mitochondrial energy metabolism, YBT maintains neurometabolic homeostasis and may alleviate oxidative stress and peripheral sensitization (Xin et al., 2015). Evidence further indicates that YBT reshapes the gut microbiota and influences systemic inflammatory signaling via the gut-brain axis, highlighting its multisystem therapeutic mechanism (Bai et al., 2021). These pharmacological findings collectively corroborate the clinical efficacy observed in this study, demonstrating that YBT is not merely an analgesic but a complex formulation capable of modulating pain, inflammation, and the neuronal microenvironment through multiple biological pathways. These findings are also broadly consistent with the 2024 Evidence-Based Guidelines for Traditional Chinese Medicine in Lumbar Disc Herniation (Qin et al., 2024), which suggest using YBT capsules alone or in combination with usual western medical therapy to relieve pain and improve physical function in LDH patients with syndrome of qi stagnation and blood stasis. Our results further support this recommendation by showing improvements in pain, function, and inflammatory markers, together with favorable short-term tolerability. Therefore, YBT can be considered a promising conservative treatment option recommended in current TCM and integrative medical practices.

This meta-analysis indicates that YBT capsules demonstrate favorable safety compared to conventional therapy. The incidence of adverse events was significantly lower in the YBT group, with most reported reactions being mild, self-limiting events such as gastrointestinal discomfort, dizziness, and rash. No serious adverse reactions or drug-related discontinuations were observed across the included studies. This suggests YBT capsules may be better tolerated than standard pharmacological therapy, potentially due to their anti-inflammatory and neuroprotective effects from the multi-component formulation. This may be clinically relevant because conventional drug therapies, particularly prolonged NSAID use, are often limited by gastrointestinal, cardiovascular, and renal safety concerns. Therefore, YBT capsule-based therapy may have potential value as an adjunctive conservative treatment option. This issue may be particularly pronounced in elderly patients, as they typically have more comorbidities and may be more susceptible to adverse effects resulting from long-term use of NSAIDs. As a complementary conservative treatment option, YBT capsule therapy may offer potential benefits for older adults. We further conducted exploratory subgroup analyses based on the mean age of the study participants. The results showed consistent improvements in VAS scores in both the ≥55 years and <55 years subgroups. Regarding adverse events, the overall trend favored YBT in both subgroups, with a more significant reduction observed in the ≥55 years subgroup. However, the differences between the subgroups were not statistically significant. Therefore, although these findings suggest that the potential benefits and tolerability of YBT are not significantly limited by the age composition of the study population, the current evidence is insufficient to support definitive conclusions regarding older adults, as the included studies did not specifically recruit older adults and did not report age-stratified outcomes. Future high-quality RCTs should strengthen adverse-event monitoring and age-stratified reporting to further clarify the clinical safety of YBT, especially in older adults.

Several key outcomes in this study, particularly VAS and IL-6, exhibited substantial statistical heterogeneity that warrants cautious interpretation. Although we employed random-effects models and subgroup and sensitivity analyses, high *I* ^*2*^ values may reflect genuine clinical and methodological diversity rather than solely random error. Baseline patient characteristics (age, symptom duration, initial pain, and functional status) varied, and most original studies did not completely report these data. YBT dosing regimens (twice daily in one trial versus three times daily in others) and treatment durations (2–4 weeks) also varied slightly. Comparison regimens and combined interventions also varied, involving different NSAIDs such as celecoxib, loxoprofen, naproxen, ibuprofen, diclofenac, and meloxicam, sometimes combined with mannitol or corticosteroids, as well as physical therapies like lumbar traction, massage, acupuncture, and TDP therapy. Although the extracted VAS values were consistent with a 0–10 point scale across studies, other aspects of VAS assessment, such as reporting details and implementation procedures, were not fully standardized. For laboratory outcomes such as IL-6, the wide variation in reported value ranges suggests that differences in assay methods, detection kits, or laboratory conditions may have affected comparability. These measurement-related differences may have contributed to the substantial heterogeneity observed and cannot be fully addressed by statistical models alone. These combined variations likely contributed to the high heterogeneity observed. To further explore potential sources of heterogeneity, we conducted subgroup analyses according to treatment duration, sample size, treatment regimen, and disease duration, as well as sensitivity analysis excluding studies with unclear randomization methods. Although the overall direction of effect remained stable across these analyses, substantial residual heterogeneity persisted, suggesting that the observed variability was likely multifactorial. Well-designed large-scale trials and advanced meta-analysis techniques are needed to clarify the relative contributions of these factors. The high heterogeneity in this review implies that our pooled estimates should be interpreted primarily as indicating the direction and approximate magnitude of overall benefit rather than precise effect sizes.

This meta-analysis systematically evaluated the efficacy and safety of YBT capsules in treating LDH. Its primary strengths include a comprehensive search strategy covering both Chinese and English databases, a rigorous selection process, and in-depth exploration of sources of heterogeneity through subgroup and sensitivity analyses. Additionally, the GRADE system was employed to assess evidence quality, enhancing the transparency and reliability of the conclusions. However, this study also has certain limitations. First, the overall methodological quality of the included RCTs was only moderate, and the certainty of evidence assessed by GRADE ranged from moderate to very low across outcomes. Key domains such as allocation concealment, blinding, and attrition were often poorly reported, so the risk of bias is likely underestimated and the observed benefits of YBT may be overestimated. In particular, findings based on evidence of very low certainty should be interpreted with caution, as the true effect may differ from the pooled estimate. Nevertheless, most trials reported comparable baseline characteristics and complete outcome data at the end of treatment, which provides some reassurance regarding internal consistency of the findings. These methodological limitations significantly undermine the reliability of the pooled estimates. The current evidence is insufficient to establish the definitive efficacy of YBT capsules. It merely suggests that they may have therapeutic effects, which require further validation through rigorously designed trials. Second, substantial heterogeneity was observed for several outcomes, particularly VAS and IL-6. This suggests that the pooled estimates may not reflect a single common effect and should be interpreted cautiously. Although random-effects models and subgroup analyses were applied, measurement-related differences could not be fully addressed statistically. VAS scores appeared comparable on a 0–10 scale, but differences in implementation or assessment timing may remain. For inflammatory markers, variation in assay methods or laboratory conditions may also have limited comparability. Third, evidence regarding safety remains limited. The included studies mainly reported adverse events and general safety observations, whereas extractable laboratory safety markers, such as liver and kidney function tests, were not available. In addition, serious complications such as gastrointestinal bleeding, cardiovascular events, or kidney injury were not directly or sufficiently reported for quantitative synthesis. Therefore, the safety conclusions of this review are based primarily on reported adverse events reflecting short-term tolerability, rather than on standardized biochemical safety monitoring or assessment of major long-term complications. Finally, all studies originated from China, potentially limiting the generalizability of findings to other populations. In summary, while our results suggest that YBT capsules may offer potential benefits in alleviating pain and controlling inflammation in LDH patients, further multi-ethnic, large-scale, well-designed multicenter randomized controlled trials are required to validate these findings, strengthen the evidence base, and support any broader clinical implementation.

## Conclusion

This meta-analysis suggests that YBT capsule-based therapy may improve pain and functional impairment in patients with LDH while reducing inflammation. The available evidence also indicates favorable short-term tolerability. These findings suggest YBT capsules may serve as an adjunct to existing anti-inflammatory analgesic treatment strategies. However, given the limitations of existing evidence, such as methodological shortcomings, these findings should be regarded as preliminary conclusions. The current evidence is insufficient to draw definitive conclusions regarding efficacy or long-term safety. Given that the current evidence base is limited to small-to-medium-sized trials conducted in China, future large-scale, multicenter randomized controlled trials with standardized outcome measures are essential to validate these benefits and establish the role of YBT capsules in international clinical practice.

## Data Availability

The raw data supporting the conclusions of this article will be made available by the authors, without undue reservation.
