# Supplementary material for: Efficacy and safety of yaobitong capsules for lumbar disc herniation: a systematic review and meta-analysis
Source: Front Pharmacol. 2026 Jun 1;17:1815867. doi: 10.3389/fphar.2026.1815867 (PMC13265297; doi:10.3389/fphar.2026.1815867)
Supplement: Supplementary file 1 [file Supplementaryfile1.docx]

**Supplementary Material**

Supplementary Table 1. Detailed PRISMA checklist.

| **Section and Topic** | **Item #** | **Checklist item** | **Location where item is reported** |
| --- | --- | --- | --- |
| **TITLE** | | |  |
| Title | 1 | Identify the report as a systematic review. | Title |
| **ABSTRACT** | | |  |
| Abstract | 2 | See the PRISMA 2020 for Abstracts checklist. | Abstract |
| **INTRODUCTION** | | |  |
| Rationale | 3 | Describe the rationale for the review in the context of existing knowledge. | Introduction |
| Objectives | 4 | Provide an explicit statement of the objective(s) or question(s) the review addresses. | Introduction |
| **METHODS** | | |  |
| Eligibility criteria | 5 | Specify the inclusion and exclusion criteria for the review and how studies were grouped for the syntheses. | Materials and methods; Inclusion criteria; Exclusion criteria |
| Information sources | 6 | Specify all databases, registers, websites, organisations, reference lists and other sources searched or consulted to identify studies. Specify the date when each source was last searched or consulted. | Materials and methods; Databases and search strategies |
| Search strategy | 7 | Present the full search strategies for all databases, registers and websites, including any filters and limits used. | Databases and search strategies; Supplementary Table 2 |
| Selection process | 8 | Specify the methods used to decide whether a study met the inclusion criteria of the review, including how many reviewers screened each record and each report retrieved, whether they worked independently, and if applicable, details of automation tools used in the process. | Materials and methods; Data extraction and quality assessment |
| Data collection process | 9 | Specify the methods used to collect data from reports, including how many reviewers collected data from each report, whether they worked independently, any processes for obtaining or confirming data from study investigators, and if applicable, details of automation tools used in the process. | Materials and methods; Data extraction and quality assessment |
| Data items | 10a | List and define all outcomes for which data were sought. Specify whether all results that were compatible with each outcome domain in each study were sought (e.g. for all measures, time points, analyses), and if not, the methods used to decide which results to collect. | Materials and methods; Inclusion criteria; Data extraction and quality assessment |
|  | 10b | List and define all other variables for which data were sought (e.g. participant and intervention characteristics, funding sources). Describe any assumptions made about any missing or unclear information. | Materials and methods; Data extraction and quality assessment |
| Study risk of bias assessment | 11 | Specify the methods used to assess risk of bias in the included studies, including details of the tool(s) used, how many reviewers assessed each study and whether they worked independently, and if applicable, details of automation tools used in the process. | Materials and methods; Data extraction and quality assessment |
| Effect measures | 12 | Specify for each outcome the effect measure(s) (e.g. risk ratio, mean difference) used in the synthesis or presentation of results. | Materials and methods; Statistical analysis |
| Synthesis methods | 13a | Describe the processes used to decide which studies were eligible for each synthesis (e.g. tabulating the study intervention characteristics and comparing against the planned groups for each synthesis (item #5)). | Materials and methods; Inclusion criteria; Statistical analysis |
|  | 13b | Describe any methods required to prepare the data for presentation or synthesis, such as handling of missing summary statistics, or data conversions. | Materials and methods; Statistical analysis |
|  | 13c | Describe any methods used to tabulate or visually display results of individual studies and syntheses. | Materials and methods; Statistical analysis; Table 1; Figures 3–9 |
|  | 13d | Describe any methods used to synthesize results and provide a rationale for the choice(s). If meta-analysis was performed, describe the model(s), method(s) to identify the presence and extent of statistical heterogeneity, and software package(s) used. | Materials and methods; Statistical analysis |
|  | 13e | Describe any methods used to explore possible causes of heterogeneity among study results (e.g. subgroup analysis, meta-regression). | Materials and methods; Statistical analysis; Subgroup Analysis |
|  | 13f | Describe any sensitivity analyses conducted to assess robustness of the synthesized results. | Materials and methods; Statistical analysis; Sensitivity analysis |
| Reporting bias assessment | 14 | Describe any methods used to assess risk of bias due to missing results in a synthesis (arising from reporting biases). | Materials and methods; Statistical analysis |
| Certainty assessment | 15 | Describe any methods used to assess certainty (or confidence) in the body of evidence for an outcome. | Materials and methods; Certainty assessment of evidence |
| **RESULTS** | | |  |
| Study selection | 16a | Describe the results of the search and selection process, from the number of records identified in the search to the number of studies included in the review, ideally using a flow diagram. | Results; Study selection; Figure 1 |
|  | 16b | Cite studies that might appear to meet the inclusion criteria, but which were excluded, and explain why they were excluded. | Results; Study selection |
| Study characteristics | 17 | Cite each included study and present its characteristics. | Results; Study characteristics; Table 1 |
| Risk of bias in studies | 18 | Present assessments of risk of bias for each included study. | Results; Quality evaluation; Figure 2 |
| Results of individual studies | 19 | For all outcomes, present, for each study: (a) summary statistics for each group (where appropriate) and (b) an effect estimate and its precision (e.g. confidence/credible interval), ideally using structured tables or plots. | Results; VAS; ODI; JOA; IL-6; IL-8; TNF-α; Adverse events; Figures 3–9 |
| Results of syntheses | 20a | For each synthesis, briefly summarise the characteristics and risk of bias among contributing studies. | Results; Quality evaluation; VAS; ODI; JOA; IL-6; IL-8; TNF-α; Adverse events |
|  | 20b | Present results of all statistical syntheses conducted. If meta-analysis was done, present for each the summary estimate and its precision (e.g. confidence/credible interval) and measures of statistical heterogeneity. If comparing groups, describe the direction of the effect. | Results; VAS; ODI; JOA; IL-6; IL-8; TNF-α; Adverse events; Figures 3–9 |
|  | 20c | Present results of all investigations of possible causes of heterogeneity among study results. | Results; Subgroup Analysis; Supplementary Figures 8–13 |
|  | 20d | Present results of all sensitivity analyses conducted to assess the robustness of the synthesized results. | Results; Sensitivity analysis; Supplementary Figures 1–7 and 14 |
| Reporting biases | 21 | Present assessments of risk of bias due to missing results (arising from reporting biases) for each synthesis assessed. | Results; Publication bias assessment; Figure 10 |
| Certainty of evidence | 22 | Present assessments of certainty (or confidence) in the body of evidence for each outcome assessed. | Results; Certainty assessment; Supplementary Table 4 |
| **DISCUSSION** | | |  |
| Discussion | 23a | Provide a general interpretation of the results in the context of other evidence. | Discussion |
|  | 23b | Discuss any limitations of the evidence included in the review. | Discussion |
|  | 23c | Discuss any limitations of the review processes used. | Discussion |
|  | 23d | Discuss implications of the results for practice, policy, and future research. | Discussion; Conclusion |
| **OTHER INFORMATION** | | |  |
| Registration and protocol | 24a | Provide registration information for the review, including register name and registration number, or state that the review was not registered. | Materials and methods |
|  | 24b | Indicate where the review protocol can be accessed, or state that a protocol was not prepared. | Materials and methods |
|  | 24c | Describe and explain any amendments to information provided at registration or in the protocol. | None reported |
| Support | 25 | Describe sources of financial or non-financial support for the review, and the role of the funders or sponsors in the review. | Funding Statement |
| Competing interests | 26 | Declare any competing interests of review authors. | Conflicts of Interest |
| Availability of data, code and other materials | 27 | Report which of the following are publicly available and where they can be found: template data collection forms; data extracted from included studies; data used for all analyses; analytic code; any other materials used in the review. | Data Availability |

*From:*  Page MJ, McKenzie JE, Bossuyt PM, Boutron I, Hoffmann TC, Mulrow CD, et al. The PRISMA 2020 statement: an updated guideline for reporting systematic reviews. BMJ 2021;372:n71. doi: 10.1136/bmj.n71. This work is licensed under CC BY 4.0. To view a copy of this license, visit <https://creativecommons.org/licenses/by/4.0/>

Supplementary Table 2. Search Strategies for Each Database

| **Database** | **Search Criteria** |
| --- | --- |
| **Pubmed** | ("Yaobitong"[Title/Abstract] OR "Yao bi tong"[Title/Abstract])  AND  ("Intervertebral Disc Displacement"[MeSH]  OR "intervertebral disc herniation"[Title/Abstract]  OR "intervertebral disk herniation"[Title/Abstract]  OR "lumbar disc herniation"[Title/Abstract]  OR "lumbar disk herniation"[Title/Abstract]  OR (intervertebral[Title/Abstract] AND disc[Title/Abstract] AND herniation[Title/Abstract]))  AND  (randomized controlled trial[Publication Type]  OR controlled clinical trial[Publication Type]  OR randomized[Title/Abstract]  OR randomised[Title/Abstract]  OR randomly[Title/Abstract]  OR trial[Title/Abstract])  NOT  (animals[MeSH] NOT humans[MeSH]) |
| **Embase** | ("yaobitong":ab,ti OR "yao bi tong":ab,ti)  AND  ('intervertebral disk herniation'/exp  OR "intervertebral disc herniation":ab,ti  OR "intervertebral disk herniation":ab,ti  OR "lumbar disc herniation":ab,ti  OR "lumbar disk herniation":ab,ti  OR (intervertebral NEAR/3 disc NEAR/3 herniation):ab,ti)  AND  ('randomized controlled trial'/exp  OR 'controlled clinical trial'/exp  OR randomized:ab,ti  OR randomised:ab,ti  OR randomly:ab,ti  OR trial:ab,ti)  NOT  ('animal'/exp NOT 'human'/exp) |
| **Cochrane Library** | ("Yaobitong":ti,ab,kw OR "Yao bi tong":ti,ab,kw)  AND  ([mh "Intervertebral Disc Displacement"]  OR "intervertebral disc herniation":ti,ab,kw  OR "intervertebral disk herniation":ti,ab,kw  OR "lumbar disc herniation":ti,ab,kw  OR "lumbar disk herniation":ti,ab,kw)  AND  (randomized:ti,ab,kw OR randomised:ti,ab,kw OR trial:ti,ab,kw) |
| **Web of Science** | TS = ( ("Yaobitong" OR "Yao bi tong")AND  ("lumbar disc herniation"  OR "lumbar disk herniation"  OR ("intervertebral disc" NEAR/3 herniation)  OR ("intervertebral disk" NEAR/3 herniation) )  AND  (random* OR trial*)) |
| **CNKI** | (腰痹通 OR 腰痹通胶囊)  AND  (腰椎间盘突出 OR 腰椎间盘突出症)  AND  (随机 OR 随机对照 OR 随机分组 OR 临床试验 OR 对照试验OR 疗效观察) |
| **Wanfang Database** | (腰痹通 OR 腰痹通胶囊)  AND  (腰椎间盘突出 OR 腰椎间盘突出症)  AND  (随机 OR 随机对照 OR 随机分组 OR 临床试验 OR 对照试验OR 疗效观察) |
| **VIP Database** | (腰痹通 OR 腰痹通胶囊)  AND  (腰椎间盘突出 OR 腰椎间盘突出症)  AND  (随机 OR 随机对照 OR 随机分组 OR 临床试验 OR 对照试验OR 疗效观察) |
| **CBM** | (腰痹通 OR 腰痹通胶囊)  AND  (腰椎间盘突出 OR 腰椎间盘突出症)  AND  (随机 OR 随机对照 OR 随机分组 OR 临床试验 OR 对照试验)  AND  文献类型：（临床研究 OR 随机对照试验）  检索年限：建库至2025年 |

Supplementary Table 3. Reporting of Yaobitong capsule product information in the included trials

| **Study** | **Year** | **Intervention product** | **Information reported in the original trial** | **Composition source used in this review** |
| --- | --- | --- | --- | --- |
| Wang et al. | 2025 | Yaobitong capsules | The study identified Yaobitong capsules as the intervention and reported product-related information including manufacturer, approval number, and specification. However, detailed botanical composition, processing, extraction, excipients, and phytochemical or quality-control information were not reported. | The full composition was supplemented from official product instructions. |
| Li et al. | 2025 | Yaobitong capsules | The study identified Yaobitong capsules as the intervention and reported product-related information including manufacturer, approval number, and specification. However, detailed botanical composition, processing, extraction, excipients, and phytochemical or quality-control information were not reported. | The full composition was supplemented from official product instructions. |
| Yan et al. | 2022 | Yaobitong capsules | The study identified Yaobitong capsules as the intervention and reported product-related information including manufacturer, approval number, and specification. However, detailed botanical composition, processing, extraction, excipients, and phytochemical or quality-control information were not reported. | The full composition was supplemented from official product instructions. |
| Xin et al. | 2021 | Yaobitong capsules | The study identified Yaobitong capsules as the intervention and reported product-related information including manufacturer, approval number, and specification. However, detailed botanical composition, processing, extraction, excipients, and phytochemical or quality-control information were not reported. | The full composition was supplemented from official product instructions. |
| Zhong et al. | 2019 | Yaobitong capsules | The study identified Yaobitong capsules as the intervention and reported product-related information including manufacturer, approval number, and specification. However, detailed botanical composition, processing, extraction, excipients, and phytochemical or quality-control information were not reported. | The full composition was supplemented from official product instructions. |
| Wang et al. | 2018 | Yaobitong capsules | The study identified Yaobitong capsules as the intervention and reported product-related information including manufacturer, approval number, and specification. However, detailed botanical composition, processing, extraction, excipients, and phytochemical or quality-control information were not reported. | The full composition was supplemented from official product instructions. |
| Su et al. | 2018 | Yaobitong capsules | The study identified Yaobitong capsules as the intervention and reported product-related information including manufacturer, approval number, and specification. However, detailed botanical composition, processing, extraction, excipients, and phytochemical or quality-control information were not reported. | The full composition was supplemented from official product instructions. |
| Ma et al. | 2018 | Yaobitong capsules | The study identified Yaobitong capsules as the intervention and reported product-related information including manufacturer, approval number, and specification. However, detailed botanical composition, processing, extraction, excipients, and phytochemical or quality-control information were not reported. | The full composition was supplemented from official product instructions. |
| Wu et al. | 2017 | Yaobitong capsules | The study identified Yaobitong capsules as the intervention and reported product-related information including manufacturer, approval number, and specification. However, detailed botanical composition, processing, extraction, excipients, and phytochemical or quality-control information were not reported. | The full composition was supplemented from official product instructions. |
| Wang et al. | 2017 | Yaobitong capsules | The study identified Yaobitong capsules as the intervention and reported product-related information including manufacturer, approval number, and specification. However, detailed botanical composition, processing, extraction, excipients, and phytochemical or quality-control information were not reported. | The full composition was supplemented from official product instructions. |
| Dong et al. | 2017 | Yaobitong capsules | The study identified Yaobitong capsules as the intervention and reported product-related information including manufacturer, approval number, and specification. However, detailed botanical composition, processing, extraction, excipients, and phytochemical or quality-control information were not reported. | The full composition was supplemented from official product instructions. |
| Wu et al. | 2015 | Yaobitong capsules | The study identified Yaobitong capsules as the intervention and reported product-related information including manufacturer, approval number, and specification. However, detailed botanical composition, processing, extraction, excipients, and phytochemical or quality-control information were not reported. | The full composition was supplemented from official product instructions. |
| Sheng et al. | 2015 | Yaobitong capsules | The study identified Yaobitong capsules as the intervention and reported product-related information including manufacturer, approval number, and specification. However, detailed botanical composition, processing, extraction, excipients, and phytochemical or quality-control information were not reported. | The full composition was supplemented from official product instructions. |
| Shao et al. | 2015 | Yaobitong capsules | The study identified Yaobitong capsules as the intervention and reported product-related information including manufacturer, approval number, and specification. However, detailed botanical composition, processing, extraction, excipients, and phytochemical or quality-control information were not reported. | The full composition was supplemented from official product instructions. |
| Gao et al. | 2015 | Yaobitong capsules | The study identified Yaobitong capsules as the intervention and reported product-related information including manufacturer, approval number, and specification. However, detailed botanical composition, processing, extraction, excipients, and phytochemical or quality-control information were not reported. | The full composition was supplemented from official product instructions. |
| Hu et al. | 2014 | Yaobitong capsules | The study identified Yaobitong capsules as the intervention and reported product-related information including manufacturer, approval number, and specification. However, detailed botanical composition, processing, extraction, excipients, and phytochemical or quality-control information were not reported. | The full composition was supplemented from official product instructions. |
| Zhou et al. | 2012 | Yaobitong capsules | The study identified Yaobitong capsules as the intervention and reported product-related information including manufacturer, approval number, and specification. However, detailed botanical composition, processing, extraction, excipients, and phytochemical or quality-control information were not reported. | The full composition was supplemented from official product instructions. |

Supplementary Figure 1. Leave-one-out sensitivity analysis for VAS


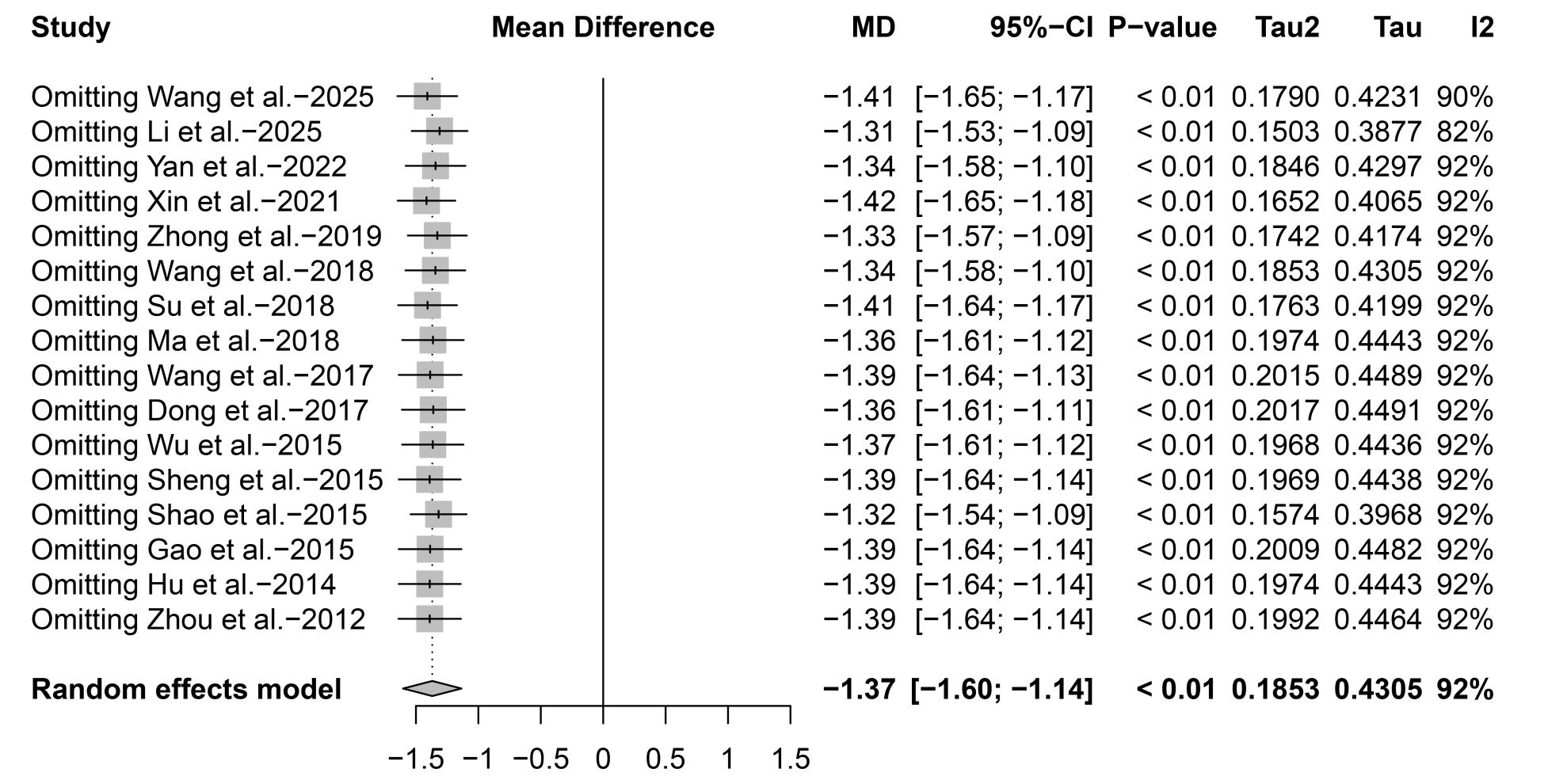


Supplementary Figure 2. Leave-one-out sensitivity analysis for ODI


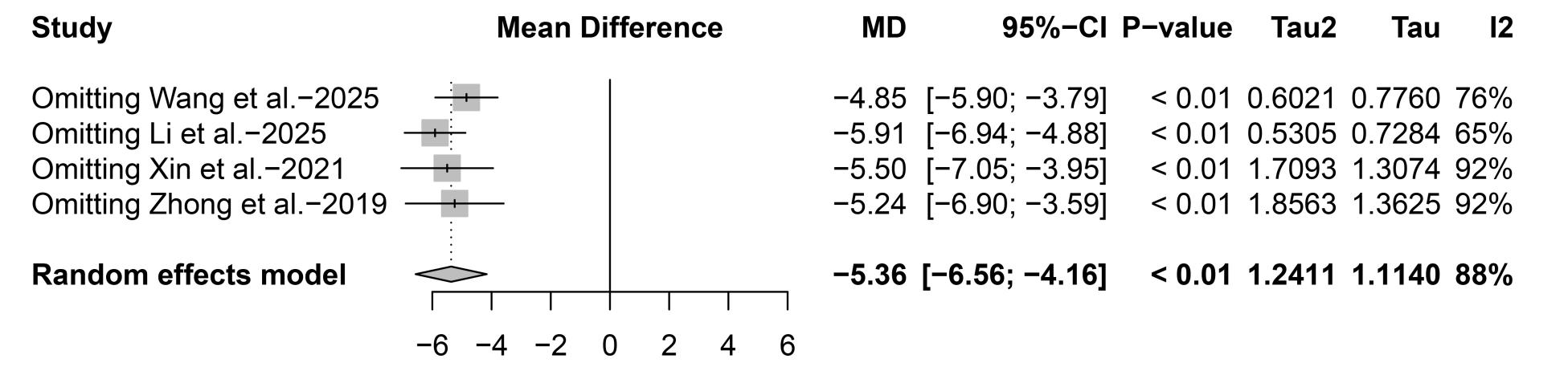


Supplementary Figure 3. Leave-one-out sensitivity analysis for JOA


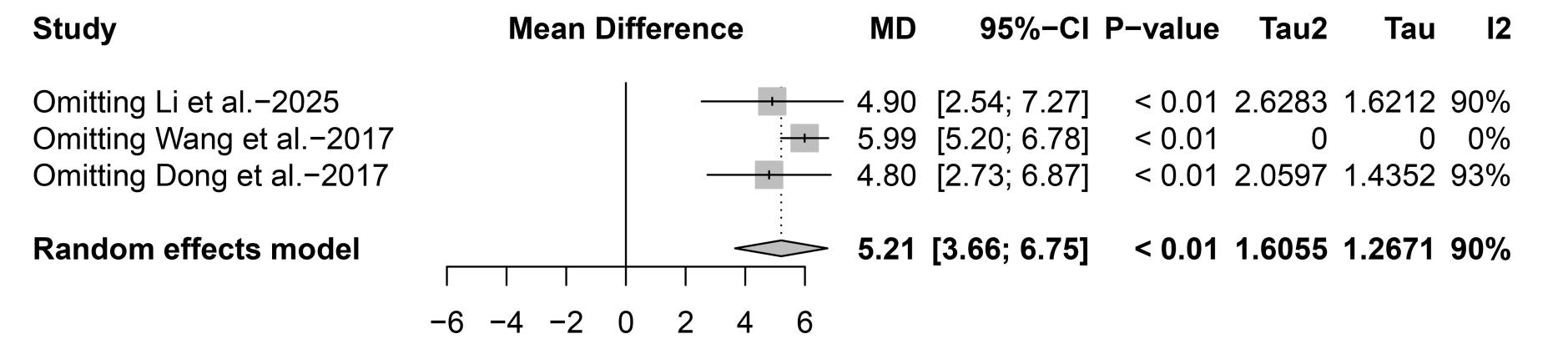


Supplementary Figure 4. Leave-one-out sensitivity analysis for IL-6


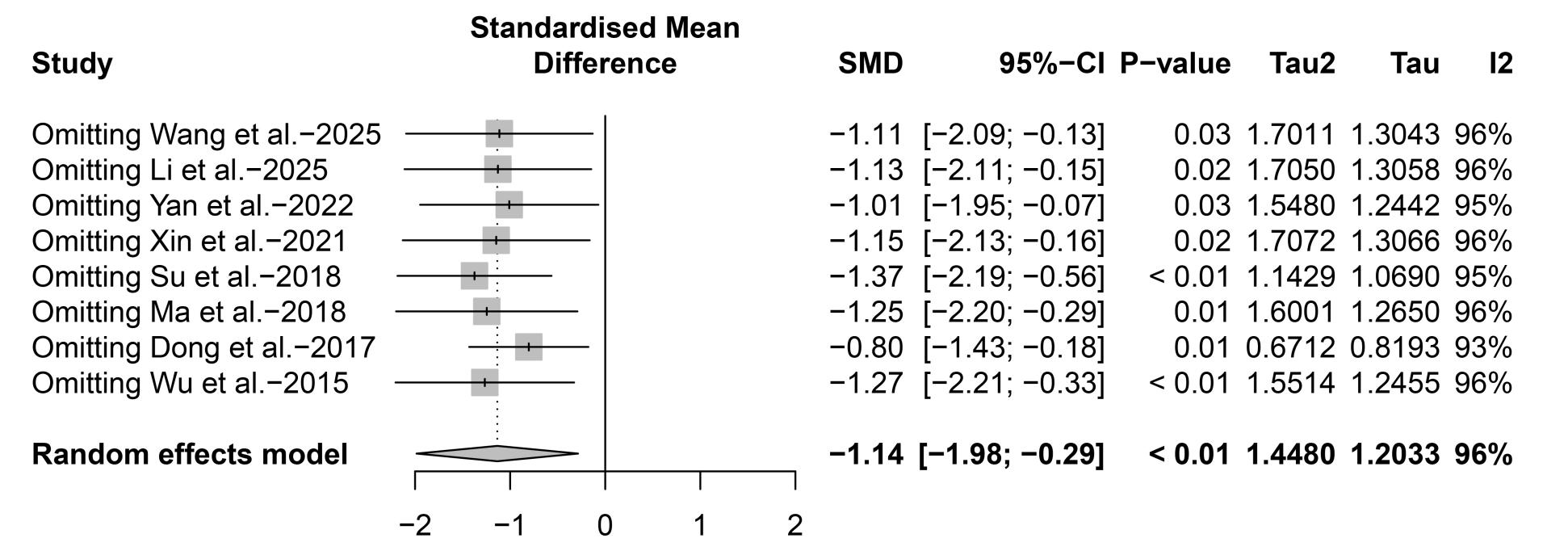


Supplementary Figure 5. Leave-one-out sensitivity analysis for IL-8


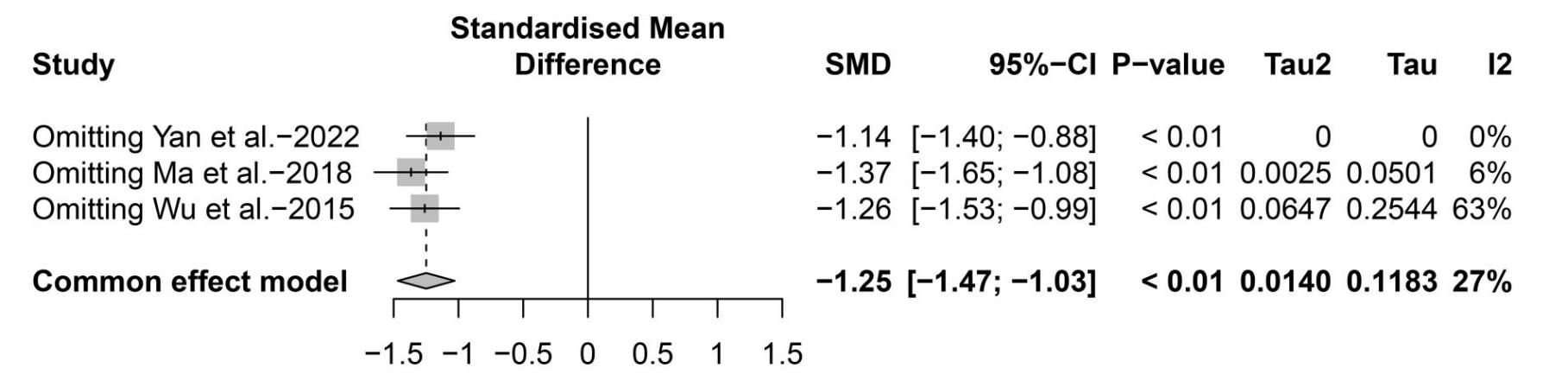


Supplementary Figure 6. Leave-one-out sensitivity analysis for TNF-α


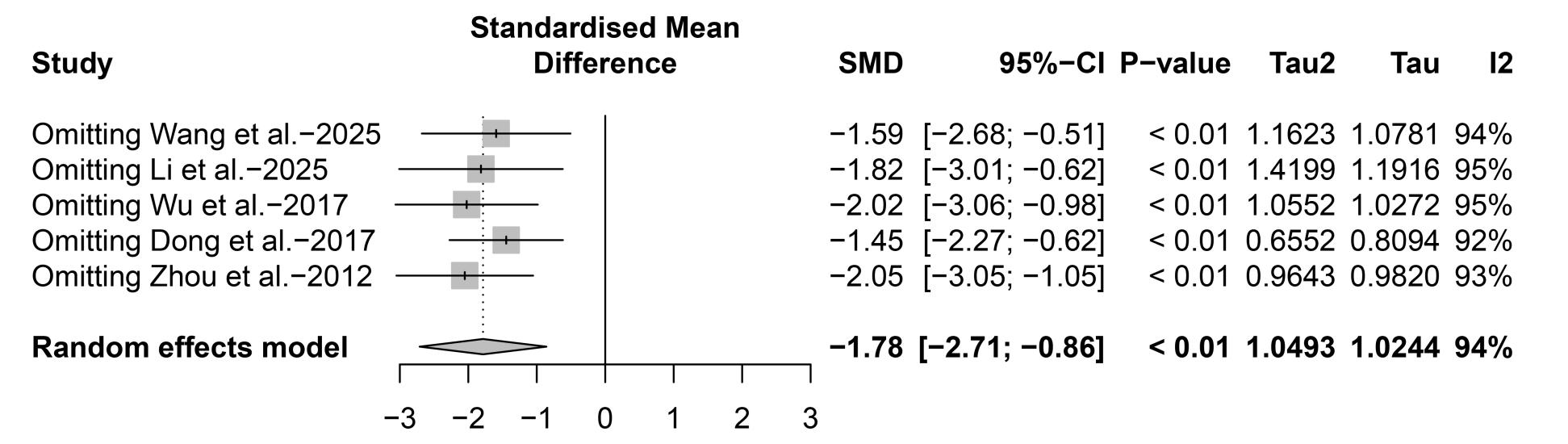


Supplementary Figure 7. Leave-one-out sensitivity analysis for adverse events


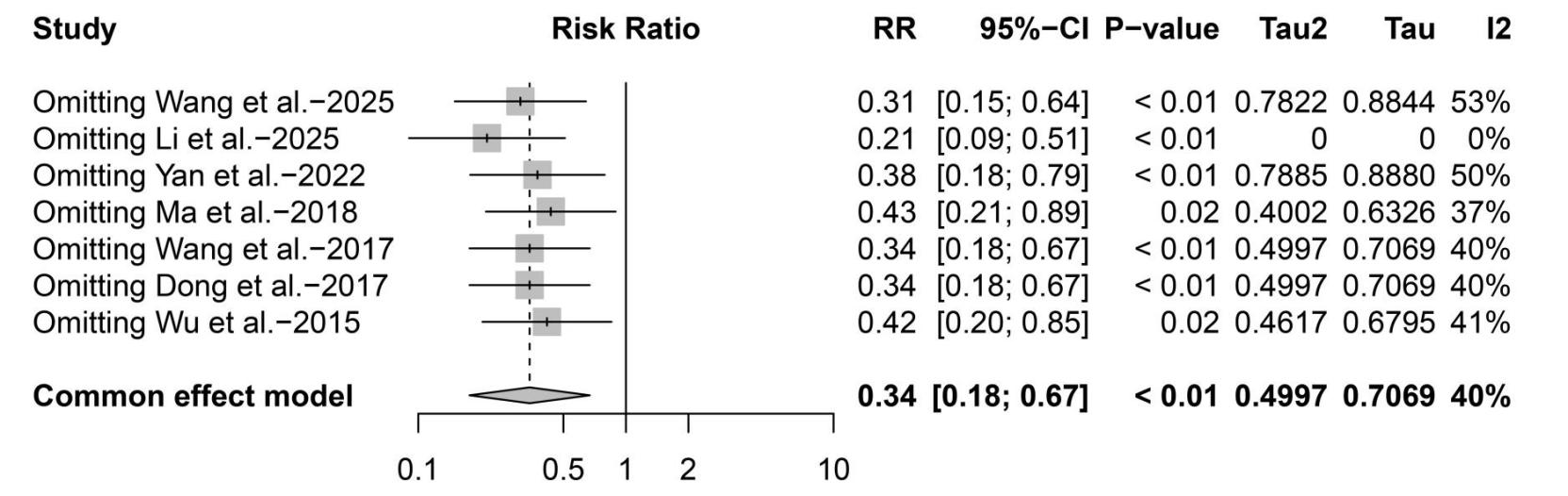


Supplementary Figure 8. Subgroup analysis by treatment duration (2 weeks, 4 weeks, 1 month) for VAS


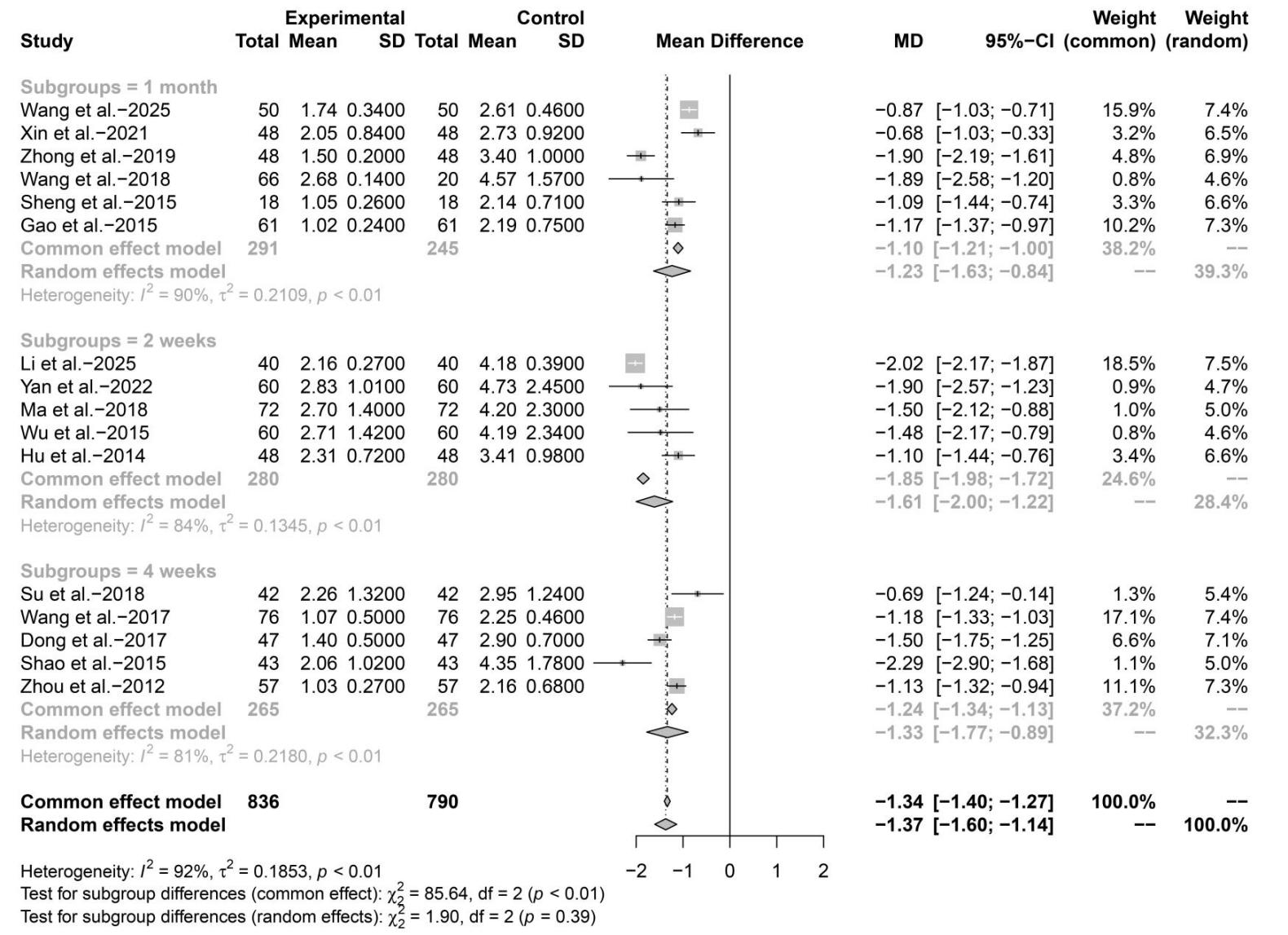


Supplementary Figure 9. Subgroup analysis by sample size category for VAS


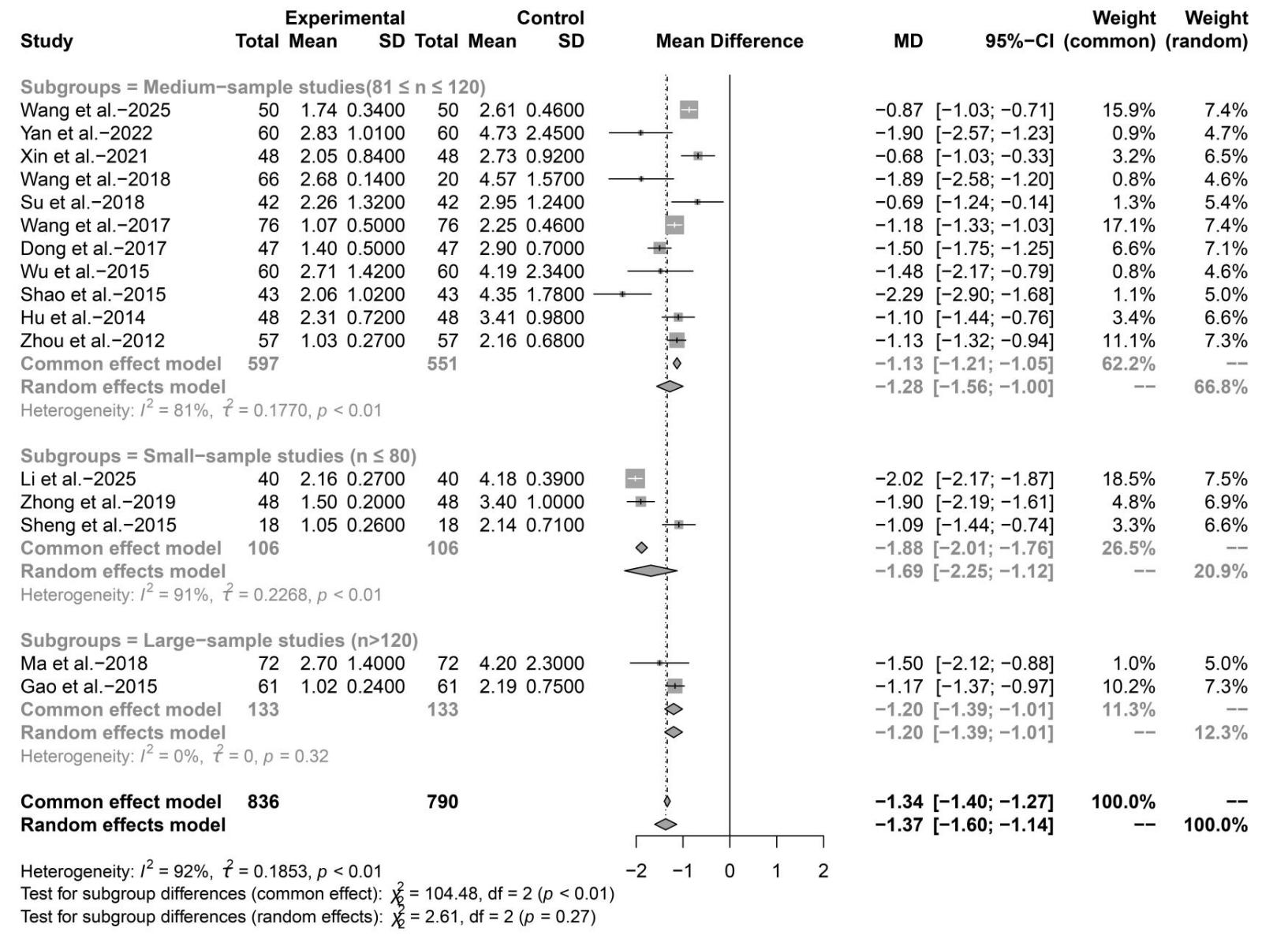


Supplementary Figure 10. Subgroup analysis by treatment regimen for VAS


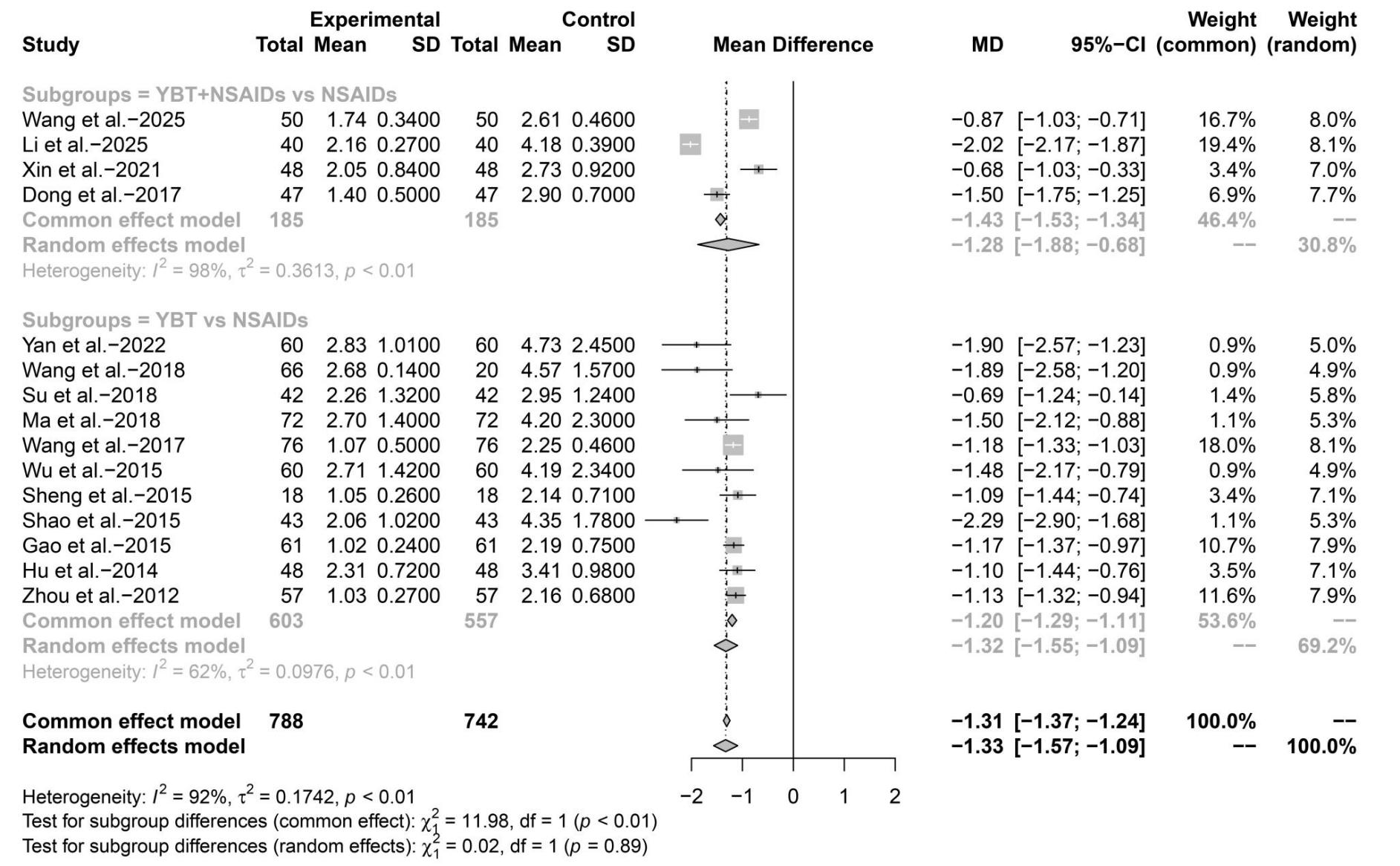


Supplementary Figure 11. Subgroup analysis by disease duration for VAS


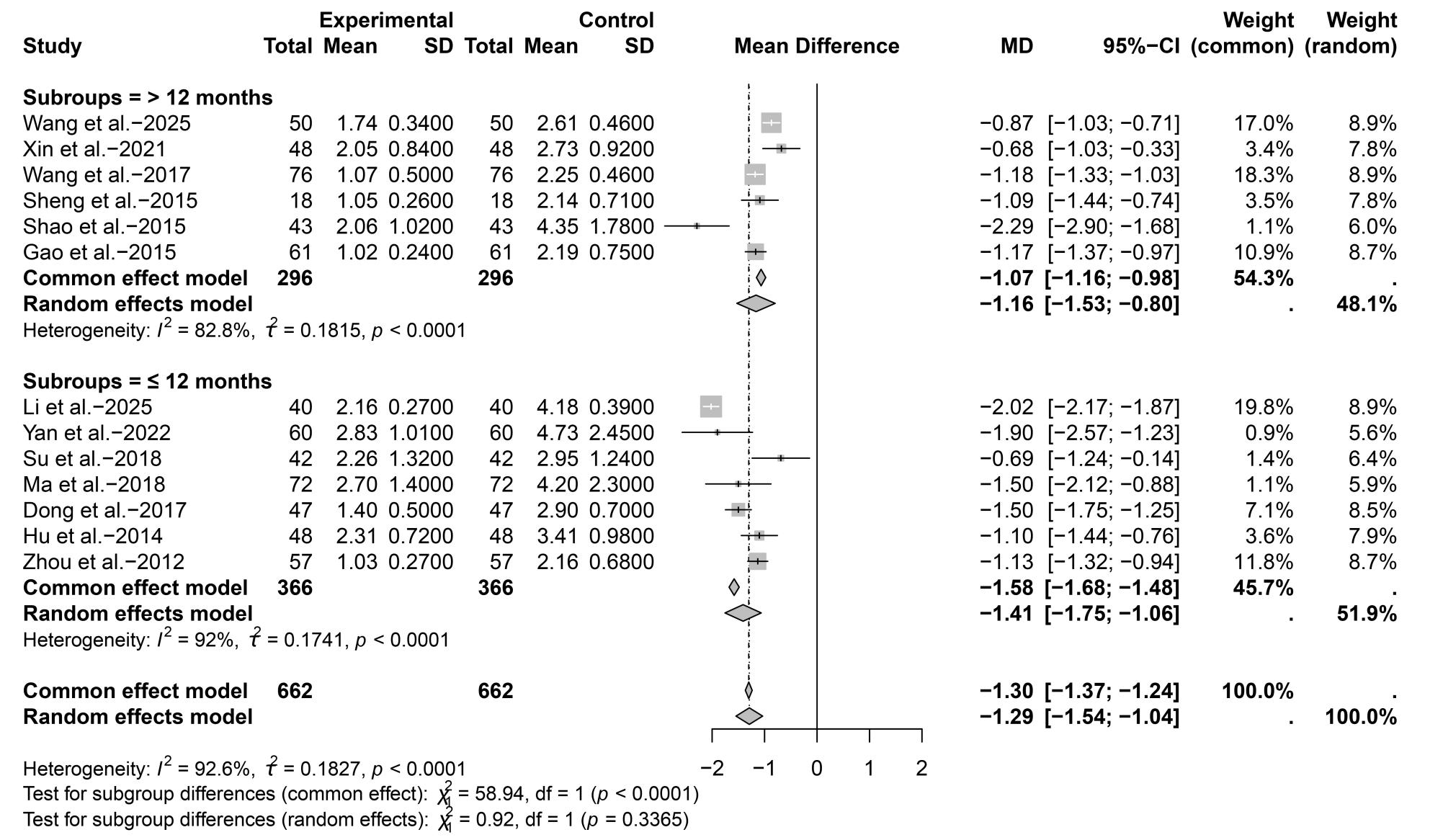


Supplementary Figure 12. Subgroup analysis by mean age for VAS


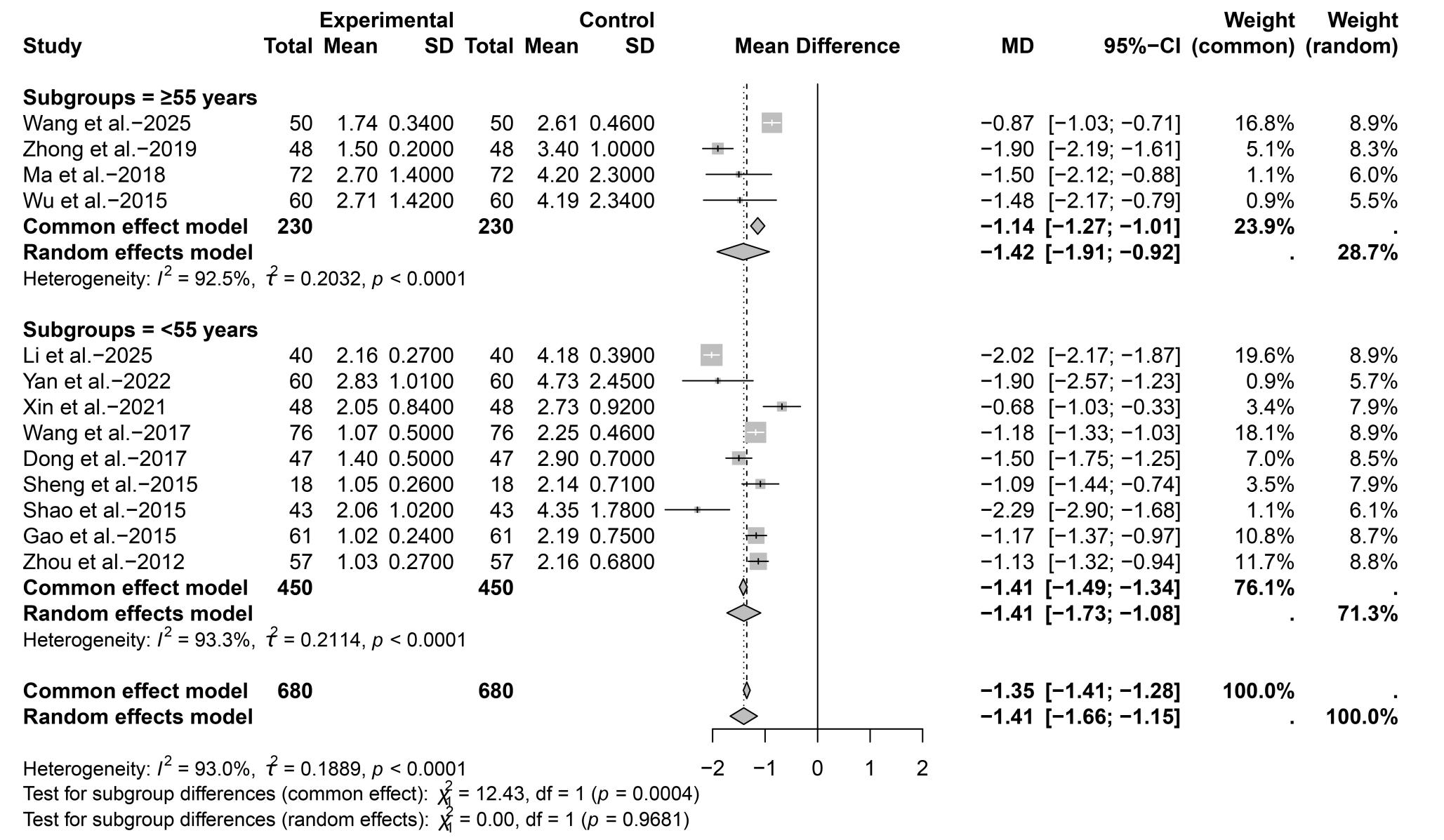


Supplementary Figure 13. Subgroup analysis by mean age for adverse events


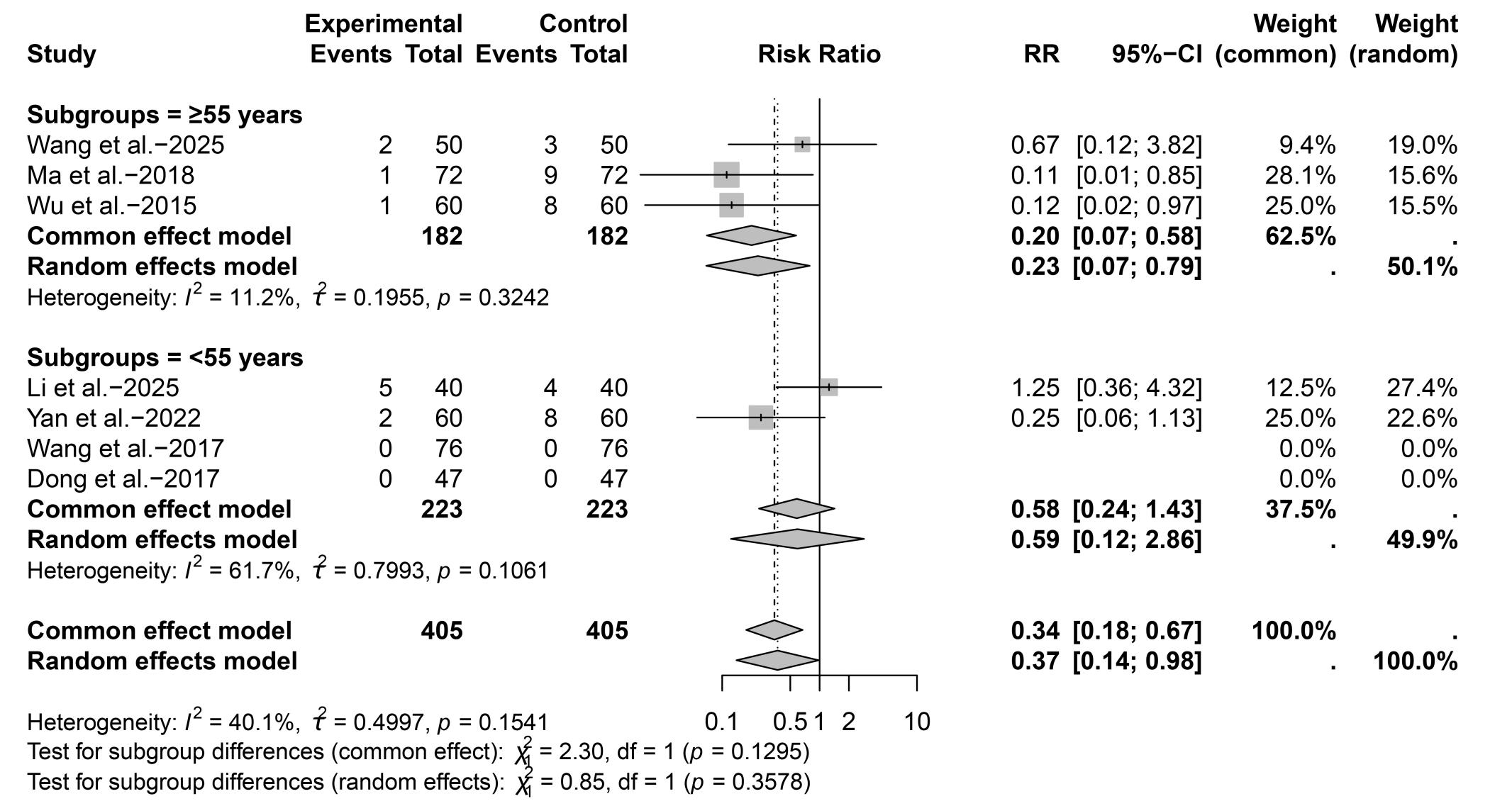


Supplementary Figure 14. Sensitivity analysis excluding studies with unclear randomization methods


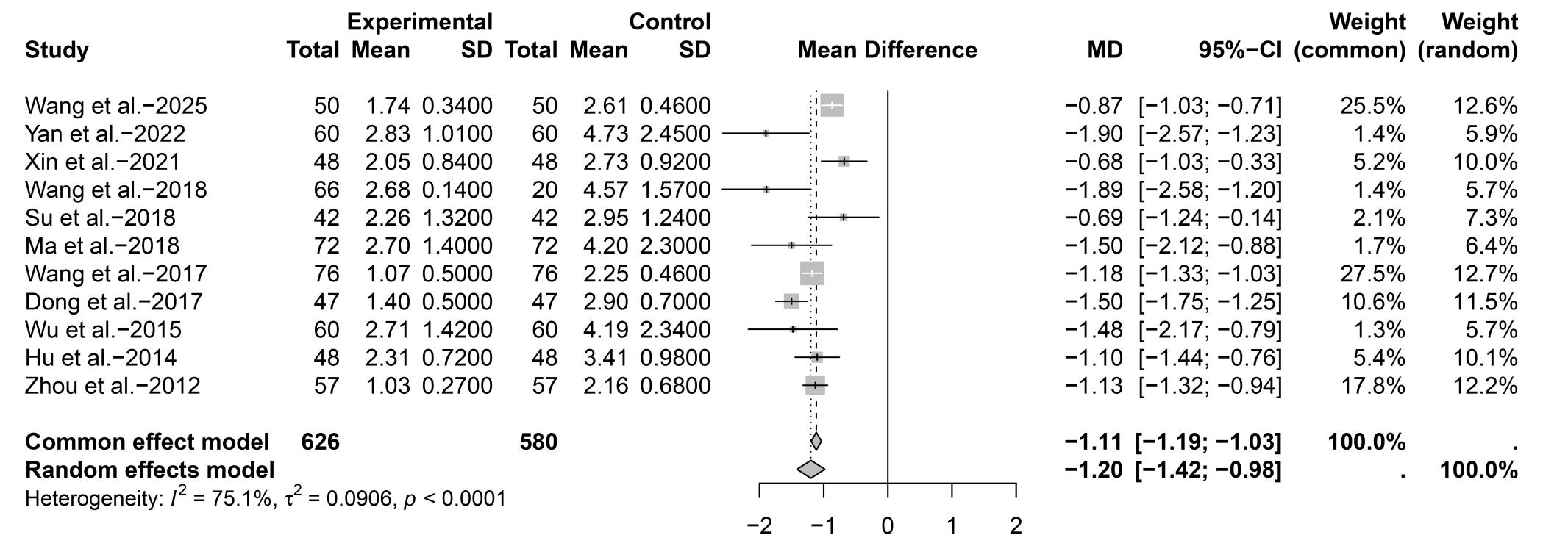


Supplementary Table 4. GRADE summary of findings and certainty of evidence.

| **Certainty assessment** | | | | | | | **№ of patients** | | **Effect** | | **Certainty** | **Importance** |
| --- | --- | --- | --- | --- | --- | --- | --- | --- | --- | --- | --- | --- |
| **№ of studies** | **Study design** | **Risk of bias** | **Inconsistency** | **Indirectness** | **Imprecision** | **Other considerations** | **Experiment** | **Control** | **Relative (95% CI)** | **Absolute (95% CI)** |  |  |
| **VAS** | | | | | | | | | | | | |
| 16 | randomised trials | serious | very serious | not serious | not serious | none | 836 | 790 | - | MD **1.37 lower** (1.6 lower to 1.14 lower) | ⨁◯◯◯ Very low |  |
| **ODI** | | | | | | | | | | | | |
| 4 | randomised trials | serious | very serious | not serious | not serious | none | 186 | 186 | - | MD **5.36 lower** (5.36 lower to 4.16 lower) | ⨁◯◯◯ Very low |  |
| **JOA** | | | | | | | | | | | | |
| 3 | randomised trials | serious | very serious | not serious | not serious | none | 163 | 163 | - | MD **5.21 higher** (3.66 higher to 6.75 higher) | ⨁◯◯◯ Very low |  |
| **IL-6** | | | | | | | | | | | | |
| 8 | randomised trials | serious | very serious | not serious | not serious | none | 419 | 419 | - | SMD **1.14 SD lower** (1.98 lower to 0.29 lower) | ⨁◯◯◯ Very low |  |
| **IL-8** | | | | | | | | | | | | |
| 3 | randomised trials | serious | not serious | not serious | not serious | none | 192 | 192 | - | SMD **1.25 SD lower** (1.47 lower to 1.03 lower) | ⨁⨁⨁◯ Moderate |  |
| **TNF-α** | | | | | | | | | | | | |
| 5 | randomised trials | serious | very serious | not serious | not serious | none | 234 | 234 | - | SMD **1.78 SD lower** (2.71 lower to 0.86 lower) | ⨁◯◯◯ Very low |  |
| **Adverse events** | | | | | | | | | | | | |
| 7 | randomised trials | serious | not serious | not serious | not serious | none | 11/405 (2.7%) | 32/405 (7.9%) | **RR 0.34** (0.18 to 0.67) | **52 fewer per 1,000** (from 65 fewer to 26 fewer) | ⨁⨁⨁◯ Moderate |  |

**CI:** confidence interval; **MD:** mean difference; **RR:** risk ratio; **SMD:** standardised mean difference
